# Wet-spinning of carbon nanotube fibers: dispersion, processing and properties

**DOI:** 10.1093/nsr/nwae203

**Published:** 2024-06-12

**Authors:** Zhicheng Yang, Yinan Yang, Yufei Huang, Yanyan Shao, He Hao, Shendong Yao, Qiqing Xi, Yinben Guo, Lianming Tong, Muqiang Jian, Yuanlong Shao, Jin Zhang

**Affiliations:** School of Materials Science and Engineering, Shanghai University of Engineering Science, Shanghai 201620, China; School of Materials Science and Engineering, Peking University, Beijing 100871, China; Beijing Graphene Institute (BGI), Beijing 100095, China; School of Materials Science and Engineering, Peking University, Beijing 100871, China; Center for Nanochemistry, Beijing Science and Engineering Center for Nanocarbons, Beijing National Laboratory for Molecular Sciences, College of Chemistry and Molecular Engineering, Peking University, Beijing 100871, China; College of Energy Soochow Institute for Energy and Materials Innovations (SIEMIS), Key Laboratory of Advanced Carbon Materials and Wearable Energy Technologies of Jiangsu Province, SUDA-BGI Collaborative Innovation Center, Soochow University, Suzhou 215006, China; Center for Nanochemistry, Beijing Science and Engineering Center for Nanocarbons, Beijing National Laboratory for Molecular Sciences, College of Chemistry and Molecular Engineering, Peking University, Beijing 100871, China; Academy for Advanced Interdisciplinary Studies, Peking University, Beijing 100080, China; School of Materials Science and Engineering, Shanghai University of Engineering Science, Shanghai 201620, China; School of Materials Science and Engineering, Shanghai University of Engineering Science, Shanghai 201620, China; Center for Nanochemistry, Beijing Science and Engineering Center for Nanocarbons, Beijing National Laboratory for Molecular Sciences, College of Chemistry and Molecular Engineering, Peking University, Beijing 100871, China; Beijing Graphene Institute (BGI), Beijing 100095, China; School of Materials Science and Engineering, Peking University, Beijing 100871, China; Academy for Advanced Interdisciplinary Studies, Peking University, Beijing 100080, China; Beijing Graphene Institute (BGI), Beijing 100095, China; School of Materials Science and Engineering, Peking University, Beijing 100871, China; Center for Nanochemistry, Beijing Science and Engineering Center for Nanocarbons, Beijing National Laboratory for Molecular Sciences, College of Chemistry and Molecular Engineering, Peking University, Beijing 100871, China; Academy for Advanced Interdisciplinary Studies, Peking University, Beijing 100080, China; Beijing Graphene Institute (BGI), Beijing 100095, China

**Keywords:** carbon nanotube fiber, wet-spinning, dispersibility, physical property-structure relationship

## Abstract

Owing to the intrinsic excellent mechanical, electrical, and thermal properties of carbon nanotubes (CNTs), carbon nanotube fibers (CNTFs) have been expected to become promising candidates for the next-generation of high-performance fibers. They have received considerable interest for cutting-edge applications, such as ultra-light electric wire, aerospace craft, military equipment, and space elevators. Wet-spinning is a broadly utilized commercial technique for high-performance fiber manufacturing. Thus, compared with array spinning from drawable CNTs vertical array and direct dry spinning from floating catalyst chemical vapor deposition (FCCVD), the wet-spinning technique is considered to be a promising strategy to realize the production of CNTFs on a large scale. In this tutorial review, we begin with a summative description of CNTFs wet-spinning process. Then, we discuss the high-concentration CNTs wet-spinning dope preparation strategies and corresponding non-covalent adsorption/charge transfer mechanisms. The filament solidification during the coagulation process is another critical procedure for determining the configurations and properties for derived CNTFs. Next, we discuss post-treatment, including continuous drafting and thermal annealing, to further optimize the CNTs orientation and compact configuration. Finally, we summarize the physical property-structure relationship to give insights for further performance promotion in order to satisfy the prerequisite for detailed application. Insights into propelling high-performance CNTFs production from lab-scale to industry-scale are proposed, in anticipation of this novel fiber having an impact on our lives in the near future.

## Teaser

This review presents an overview of advancements in wet-spinning techniques for fabricating carbon nanotube fibers. The relationship between fiber structure and performance, insights into the production of structure-function integrated carbon nanotube fibers from laboratory scale to industrial scale, along with their applications has been also elaborated.

## INTRODUCTION

High-performance fibers are generally designated as macroscopic one-dimensional materials with preeminent mechanical [1–5] and other properties, such as remarkable thermophysical [6–10], electrical [11–13] and biological properties [14–17], which are ubiquitously deployed in airplanes, space vehicles, as well as sports and leisure goods. Over the past century, there has been a rapid escalation of high-performance fibers [18]. For instance, Kevlar fiber, ultrahigh molecular weight polyethylene (UHMWPE) and carbon fibers (CFs) have been in vogue since their invention. These high-performance fibers have great advantages combining high strength and modulus with light weight, which transcend traditional metal and alloy wires. However, when compared with metallic materials, the electrical and thermal conductivity of these fibers are relatively inferior according to their polymeric chemical structure, which engenders constrained application scenarios. Thus, developing lightweight and ultra-strong fibers with electrical and thermal conductive features is a promising direction for research [19–21].

As a one-dimensional nano-material, CNTs can be regarded as a rolled-up graphene sheet with robust *sp^2^* bonds between adjacent honeycomb carbon atoms. Due to their unique one-dimensional atomic lineage structure and super-strong *sp^2^* covalent bonding, CNTs are endowed with excellent mechanical and transport properties, including tensile strength over 100 GPa, Young's modulus over 1 TPa [22,23], thermal conductivity of 3500 W m^–1^ K^–1^ [24] and electrical conductivity surpassing 2 × 10^7^ S m^–1^, respectively [25]. When assembling CNTs into fibrous configurations, the derived fibers could transfer the CNTs intrinsic features to the macroscale fibers. For instance, CNTFs demonstrate significantly enhanced knot efficiency and flexibility than CFs. Superior electrical and thermal conductivity are also demonstrated ascribed to the CNTs conjugated backbone. As a result, CNTFs demonstrate promising applications from military equipment and wearable intelligent device to specific space elevator cable, which requires a strength to mass ratio up to 48.5 GPa g^−1^ cm^−3^ [26].

In general, there are three representative strategies for producing CNTFs, including array spinning from drawable CNTs vertical array [27–30], direct dry spinning from floating-catalyst chemical vapor deposition (FCCVD) [31–34], and wet-spinning of high-concentration CNTs dispersion [19,20,35,36]. Mechanical properties of CNTFs are closely contingent on individual CNT aspect ratios (length divided by diameter, L/d) [37,38], in which long CNTs could offer luxuriant intertube friction to enhance the tensile strength. According to the long constituent CNTs (∼0.1–1 mm), direct dry spinning from FCCVD and array spinning could generate a distinguishing mechanical strength, ∼2 to 10 GPa. However, the relatively random arrangement and moderate packing density causes deterioration of both thermal and electrical conductivities of the derived CNTFs [21,39]. The thermodynamically stable and highly concentrated CNTs dispersion is the prerequisite for preparing high-performance wet-spinning CNTFs. The mechanical property of CNTFs is mainly determined by the intertube friction, which boils down to the aspect ratio, purity of CNTs source and packing-density, and orientation of the derived CNTFs. Thus, the rheological properties of CNTs liquid crystal (LC) dopes, wet-spinning parameters of spinneret nozzle, drafting and post-treatment process has been systematically optimized to enhance the packing-density and orientation of CNTFs [40–42]. Although the CNTs with a length of <10 μm (∼2–10 μm length) manifest an inferior L/d ratio than ultra-long CNTs, the remarkably enhanced dispersibility could significantly improve the degree of arrangement ordering within both liquid LC domains and concrete fiber while suppressing the CNTs aggregation [43,44]. Afterwards, post-treatments, such as drafting and thermal annealing, could further promote the CNTs alignment and strengthen the interfacial interaction between individual CNTs. Thus, based on the above synergistic effects of high-quality CNTs source, parameter optimization of wet-spinning and post-treatment, the derived wet-spinning CNTFs could demonstrate superior load transfer between tubes, contributing to great mechanical properties alongside preferable thermal and electrical conductivity [41].

Although some reviews [25,45–51] have included the description of CNTFs fabrication and application, a comprehensive summary to give detailed insights into CNTFs wet-spinning process is still missing. Herein, we not only render the development course of wet-spinning CNTFs, but also summarize the wet-spinning requisite parameters that occur in sequence. First, two types of dispersion mechanisms through non-covalent adsorption and charge transfer will be mainly discussed. Second, understanding of the diffusion-controlled phase separation process with insights into the kinetics and thermodynamics during the coagulation bath will be analyzed. Moreover, two commonly utilized post-treatment strategies, drafting and thermal annealing will be also dissected. Resuming the thread of a panoramic discourse of detailed CNTFs wet-spinning process, their mechanical, electrical, and thermal properties will also be discussed with a special pivot on the correlations between multilevel structures and macro-scale properties. Finally, we provide some perspectives on bridging the gap between individual CNT and macro-scale CNTFs, in combination with large-scale production and potential applications.

## GENERAL INTRODUCTION OF CNTFS WET-SPINNING PROCESS

### Development history of wet-spinning CNTFs

**Figure 1.**
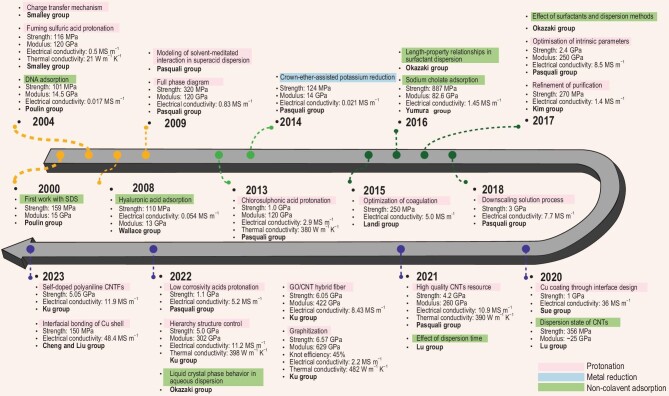
The overall development history of wet-spinning CNTFs. The different background colors stand for varied CNTs dispersion approaches [19,20,36–38,40–42,53–73].

Since the initial discovery of CNTs by Iijima in 1991 [52], their remarkable thermal, mechanical, and electrical properties have sparked tremendous interest in synthesizing macroscopic assemblies that maintain these exceptional characteristics. In Fig. 1, we have summarized the developmental progress over the past two decades. The trajectory of wet-spinning CNTFs has witnessed various stages, beginning with the successful fiber spinning, evolving through the enhancement of spinning conditions and interfacial modulation to achieve property optimization.

The commencement of utilizing wet-spinning to fabricate macroscopic CNTFs can be traced back to 2000. Vigolo *et al.* [36] first dispersed single-walled CNTs (SWCNTs) in an aqueous solution with sodium dodecyl sulfate (SDS), then obtained CNTFs with tensile strength of 159 MPa and modulus of 15 GPa. However, the electrical conductivity of the derived CNTFs was inferior due to the incorporation of polyvinyl alcohol (PVA) in the coagulation bath, which was then embedded into the CNTFs and impeded efficient electron transport. Then, in a bid to maintain the intrinsic advantages of CNTs resources, polymer-free coagulation bath was investigated. Bio-amphiphilic substances such as DNA [53] and hyaluronic acid (HA) [54] were employed as dispersants. The as-fabricated CNTFs demonstrated mechanical properties on par with those coagulated in PVA, but with a greatly augmented electrical conductivity in the order of 10^4^ S m^–1^.

Hereto, further property promotion of surfactant-based CNTFs is realized under the interpretation of the relationships between CNTs length and the property of the ensuing fibers [38]. Then, after the alleviation of truncation effect aroused by sonication, tensile strength and electrical conductivity of the as-prepared CNTFs reach GPa and MS m^–1^ level [19,55].

The charge transfer dispersion phenomenon induced by both superstrong acids and alkali metals are also disclosed. Meanwhile, the tensile strength of CNTFs spun from fuming sulfuric acid dispersed CNTs dope is comparable to that of fibers spun from the amphiphilic surfactant assisted CNTs dispersion, which are at the level of 100 MPa [36,53,57,73]. However, the modulus which is intensively correlative to intertube alignment of the fuming sulfuric acid dispersion triggered CNTFs is 10 times higher. Moreover, superacid doping completed as-spun CNTFs with electrical conductivity an order of magnitude higher. In the following decade, research work mainly pivoted on the liquid phase behavior [54,59] and solvent-mediated interaction of superacid-CNTs dispersions [58]. The promoted studies have facilitated the technological development of wet-spinning, leading to a performance breakthrough with a tensile strength up to 1 GPa, a modulus up to 120 GPa, an electric conductivity of 2.9 MS m^−1^, and a superior thermal conductivity up to 635 W m^−1^ K^−1^ after iodine-doping [19].

Further studies have emphasized the intrinsic characteristics of CNTs including diameter, wall number, length, graphitic structure, and purity. The results corroborate the significance of CNTs aspect ratio and purity [37,38,60]. As a result, the mechanical properties of CNTFs assembled with a large aspect ratio could even reach 2-fold values (tensile strength of 2.4 GPa, Young's modulus of 250 GPa) compared to the benchmark performance obtained in 2013 [37].

Recently, research efforts have mainly concentrated on the extrinsic parameters throughout the spinning processes including spinneret morphology [74], extensional drawing ratio [44] and coagulation process [60,75] in order to minimize inter-bundle and intra-bundle voids [41]. Thus achieving a series of benchmarking properties, such as a tensile strength of 5.0 GPa, Young's modulus of 302 GPa, which even approaches commercial CFs, such as K13D2U, IM9 and T700. Other parameters, such as knot efficiency (85%) and electrical conductivity (11.2 MS m^–1^) far exceed that of CFs.

Concomitant with the exploration of wet-spinning extrinsic parameters, regulation of interfacial properties with enhancing the intertube interaction is also a promising avenue for preparing high-performance CNTFs. For instance, copper surface encapsulation has been utilized to facilitate charge carrier transportation for achieving an electrical conductivity of 36 MS m^–1^ [64], while heat treatment to form a close-packed graphitic domain resulted in a tensile strength of 6.57 GPa [42]. In addition, intermediate mediator introduction *via* graphene oxide hybridizing to regulate the viscoelasticity and fill the voids gave a 6.05 GPa tensile strength [66].

### Basic wet-spinning process of CNTFs

**Figure 2.**
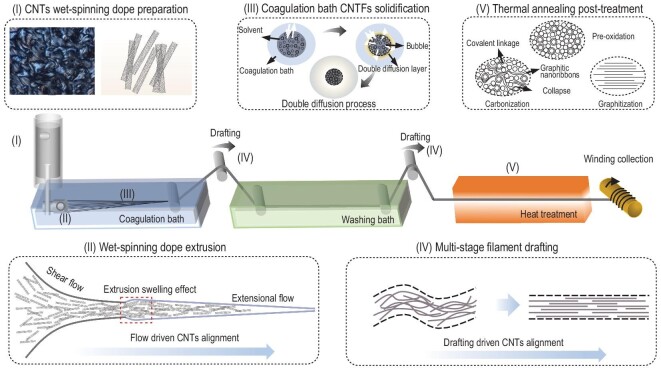
Schematic illustration of the detailed wet-spinning CNTFs process, including (Ⅰ) CNTs wet-spinning dope preparation. Adapted with permission from Ref. [59]. Copyright 2009 Springer Nature, (Ⅱ) wet-spinning dope extrusion, (Ⅲ) CNTs filament solidification in coagulation bath, (Ⅳ) multi-stage drafting, and (Ⅴ) thermal annealing post-treatment.

Figure 2 schematically illustrates the general wet-spinning process for preparing CNTFs. Overall, the structure and derived properties of CNTFs mainly depend on the following five critical factors, (I) homogeneity of the wet-spinning dopes, (II) fluid rheologic features and spinning extrusion behavior, (III) dual diffusion process in the coagulation bath, and post-treatments such as (IV) multi-stage filament drafting and (V) thermal annealing [76,77]. Among these procedures, the kinetic and thermodynamic processes determine the conformational changes in CNTFs, which in turn control the densification, orientation and even crystallinity.

As shown in Region I, high-quality CNTs dispersions exhibit a distinct LC phase behavior. The quality of the dispersion depends on the following three aspects, the intrinsic structure of CNTs, the dispersion mechanism of the selected solvent and the dispersion concentration.

To optimize the CNTs quality, pre-treatments, such as high temperature annealing and acid washing, *etc.*, are essential for removing any impurities [37,78]. The presence of residual catalyst and amorphous carbon in the pristine CNTs could significantly affect the CNTs dispersibility, which subsequently plays a remarkable role in determining their rheological properties as well as fiber structure and performance.

The proper solvent is crucial for preparing highly concentrated CNTs dispersion with ordered LC phase behavior. One critical factor in determining the wet-spinning kinetics and thermodynamics is the interaction between CNTs and the solvent molecules. The formation of a stable CNTs dispersion can be achieved by using a variety of non-covalent adsorbents (SDS, *etc.*) and strong acids (fuming sulfuric acid, chlorosulfonic acid (CSA), methanesulfonic acid, *etc.*). The mechanism will be described in detail in Section Spinning dope preparation strategies. Due to the sturdy van der Waals force inside individual CNT bundle [79], it's quite challenging to disperse CNTs individually. As a non-thermal solvent, CSA could generate an effective electrostatic repulsive force to counteract the van der Waals interactions between individual CNT *via* a protonation effect, which is conducive to forming the ordered nematic phase LC domains in a sufficient concentration [80].

The rheological property, such as viscosity, has a significant effect on the spinnability [44]. Spinnability is the deformability of a fluid subjected to a steady stretching operation, i.e. the ability of a fluid to form fibers under tension. The spinnability is essentially rheological-dependent, highlighting the significance of dispersion viscosity. Mukai K *et al.* [55] have reported that at a shear rate of 0.1 s^–1^, a critical viscosity of over 10 Pa·s is needed to produce CNTFs. Empirically, some viscosity-related parameters can be used to determine spinnability, such as the structural viscosity index ($\Delta \eta )$ and elongational viscosity (${{\eta }_e}$). These two parameters are negatively correlated with spinnability, in which the larger value leads to the worse spinnability. To obtain the desired viscoelasticity, the appropriate length-to-diameter ratio as well as the solid content of CNTs dispersion should be systematically optimized. The concentration of spinning dope will distinctly slow down the dual diffusion rate while densifying the CNTFs. However, the excessive viscosity of the spinning dope will also lead to degraded spinnability, which severely blocks the spinneret and hinders continuous wet-spinning. Therefore, it is necessary to select appropriate spinning concentration and proper quality of CNTs to manipulate the wet-spinning dope rheological properties.

As shown in Region II, the liquid phase wet-spinning dope will transform into gel-state filament after being extruded from the spinneret. During this process, the randomly distributed LC domain of CNTs form into a highly oriented configuration, according to the driven alignment of flow and shear force. The spinneret shape and channel configuration are the key factors to achieve controllable shear fluid behavior with proper fiber configuration and orientation. They also determine the spinning dope fluid distribution as it passes through the spinneret aperture. Different geometry parameters, such as aperture size, shape, and tunnel arrangement significantly affect the fluid behavior, including fluid velocity distributions. Uniform velocity distribution promotes uniform fluid shearing, which contributes to the consistent alignment of individual CNT in CNTFs [74,75]. In general, the spinneret inner diameter size plays a pivotal role in determining the fluid viscosity gradient.

The coagulation bath is another crucial step in determining the configuration of the final derived CNTFs (Region III in Fig. 2). The coagulation step is essentially a phase separation process, which is controlled by coagulating bath and spinning solvent dual diffusion [81]. The mass transfer rate between solvent and non-solvent (coagulant) determines the coagulation rate and final morphological structure of CNTFs. The solidification process involves not only the dual diffusion at the CNTs solvent/coagulant liquid interface but also includes chemical reaction processes [41,60], which form a diffusion layer (yellow area in Region Ⅱ) on the surface of the fiber to facilitate fiber solidification. Several key parameters such as the composition, density, concentration, and temperature of the coagulation bath play a decisive role [82]. When the mass transfer rate of the coagulant is lower than that of the solvent, the fiber will rapidly contract into a dense structure. Meanwhile, the bubbles generated inside the fiber will be excreted to the fiber surface, further increasing the packing density. When the coagulant diffusion rate is higher than the solvent diffusion rate, the derived CNTFs will solidify before the solvent is fully diffused out from the filament gel, which will lead to fiber diameter expansion. After the nascent filament deviated from the coagulant, this process tends to leave many pores inside the derived CNTFs. In addition, the bath temperature also has a great influence on the diffusion rate and thus-derived fiber porosity. For example, an elevated temperature tends to accelerate the non-solvent diffusion rate, which will lead premature fiber solidification before being able to form into a compact configuration. In addition, regulation of the coagulation bath is associated with other factors, such as shear rate, spinning speed, concentration gradient and mixing entropy, which affect the migration and phase separation processes of solvent and non-solvent during the coagulation process [82]. The diffusion kinetics and fiber formation thermodynamics studies help to control the solidification process during CNTFs wet-spinning.

The initial fiber obtained after the coagulation process usually exhibits structural defects such as CNTs insufficient entwining orientation, and porosity. Therefore, the drafting treatment in the post-treatment process (Region IV) has been designed to remove the structural defects of CNTFs [41]. Afterwards, the heat treatment process (Region V) can further repair the structural defects, eliminating the residual stress to improve the orientation and crystallinity of the final derived CNTFs to further enhance the inter-nanotube covalent bonding and corresponding modulus thermal conductivity features [42]. The heat treatment generally includes pre-oxidation, carbonization, and graphitization processes. During the fiber annealing process, the intact CNTFs will initially undergo inter-tubular covalent crosslinking, graphitic nanoribbons formation, and tube collapse during the carbonization process [83].

## SPINNING DOPE PREPARATION STRATEGIES

It has been estimated that if CNTs bundle could eliminate all the negative effects from catalyst particles, voids and tube entanglements, SWCNTs fibers with 2 nm in diameter could reach the ideal tensile strength of 70 GPa [18]. Recently, Wei *et al.* [84] have already validated the mechanical property potential by assembling centimeters long CNTs into bundles, which reach a tensile strength over 80 GPa, equivalent to 10 times of the strongest commercial fiber today.

Yet successful manufacturing of high-performance CNTFs begins with understanding and controlled preparation of high-concentration CNTs dispersion. The prerequisite for achieving highly aligned structure in CNTFs is to leverage pre-aligned precursors, i.e. CNTs LC phase in the wet-spinning dope. There is adequate evidence to prove that the CNTFs orientation is proportional to the degree of CNTs LC phase [63]. This phenomenon highlights the indispensable role of CNTs LC phase for preparing high-performance CNTFs.

**Figure 3.**
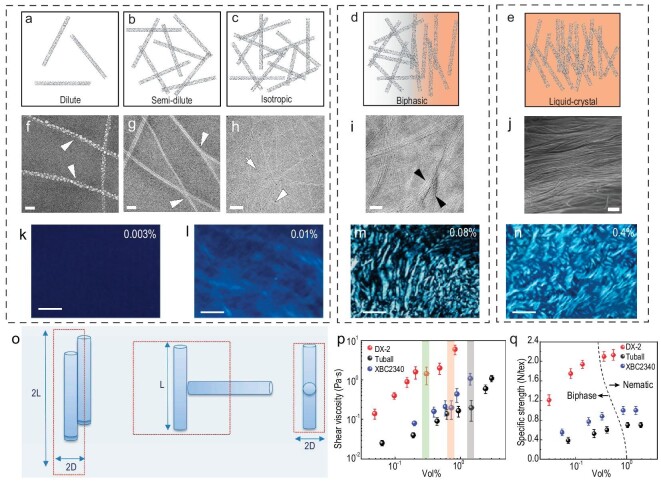
Schematic illustration of the dispersion states of CNTs and corresponding cryo-EM images depending on increasing concentrations from dilute (a and f), semi-dilute (b and g), isotropic (c and h), biphasic (d and i) to LC (e and j). Birefringence patterns of different concentration regions. Dilute and semi-dilute regions corresponding to (k). (l) Shows the birefringence of the boundary state between isotropic phase and biphase. Birefringence of biphase and LC correspond to (m and n), respectively. (o) The exclusive volume of 1D particles. (p) Phase transition concentration from biphase to nematic phase determined from shear viscosity-volume fraction curves and (q) correlation between spinning dope LC phase with the specific strength of the ensuing fibers. Scale bars: 50 nm (f–i), 500 nm (j) and 100 μm (k–n). (f–j) Adapted with permission from Ref. [90]. Copyright 2017 American Chemical Society. (k–n, p–q) Adapted with permission from Ref. [41]. Copyright 2022 Elsevier. (o) Adapted with permission from Ref. [91]. Copyright 2017 John Wiley and Sons.

To realize the high-performance CNTFs fabrication with perfect orientation, the CNTs LC phase formation mechanism should also be studied systematically. Onsager [85] proposed a statistical thermodynamic theory of LC phase transition of rod-like molecules in colloidal particles which was further enriched by Flory [86]. The entropy involved in 1D particles (CNTs and rigid-rod like polymers) suspensions can be categorized into two inverse-related forms, namely orientational entropy (depicted by the alignment of particles) and translational entropy (occupied by the anisotropic particles excluding the spatial center of an extraneous particle). The translational entropy is also named as exclusive volume entropy indicating to a certain volume scaling with $d{{l}^2}$, where *d* and *l* refer to the diameter and length of CNTs, respectively [87]. It can be seen from Fig. 3o, at two extreme circumstances, parallel configuration has minimal excluded volume which equals to the maximum translational entropy. However, orientational entropy is reduced simultaneously to the nadir. When individual CNTs are approaching perpendicular configuration, there is an inverse change that translational entropy diminishes while orientational entropy augments. Thermodynamically, above a certain critical concentration, CNTs incline to form an orientationally organized form in the dispersion for retaining the lowest Gibbs free energy. The increment of translational entropy offsets the deficit of orientational entropy, resulting in a favorable free energy reduction. Statistically speaking, the dimensionless Onsager excess free energy *f* for a dispersion is correlated with CNTs volume *V* and diameter *d*. Meanwhile, poly-dispersity length *L* is depicted by a distribution function $N( l )$ referring to the rods number in the dispersion system. Relative length *l* is defined as $L/{{L}_0}$, in which ${{L}_0}$ is an arbitrary reference length. Then decouple *f* can be written as equation (1):


(1) \begin{eqnarray*}
f &=& \frac{{bF}}{{V{{k}_{\boldsymbol{B}}}T}} \sim \int c (l) [ {\ln c (l) - 1} ] dl\\
&&+ \int c (l) \omega (l)dl\\
&&+ \int\!\!\!\int c(l)c (l{^{\prime}})ll^{{\prime}} [{\rho ( {l},l^{\prime}) + \lambda( {l},l^{\prime} )}] dldl^{\prime},\\
\end{eqnarray*}

where the reference volume *b* is defined as $\pi dL_0^2/4$ and the dimensionless rod distribution function $c( l )$ is defined as $bN( l )/V$. Three entropic terms consitute the formula in the free energy description. The first term is derived from the ideal free energy of polydispersed rigid-rods while the second term contains the entropy intertwined with orientation. Then $\omega ( l )$ could be expressed as equation (2):


(2) \begin{eqnarray*}
\omega ( l ) = \int \psi ( {l,u} )\ln [ {4\pi \psi ( {l,u} )} ]du.
\end{eqnarray*}

It describes the normalized angular distribution function for rods with relative length *l*. In addition, angular distribution is dictated by the rod orientation, while orientation described by the unit vector *u* is given as $\psi ( {l,u} )$. The uniform and disordered CNTs isotropic phase manifests the smallest value, i.e. $\psi = 1/4\pi $. Excluded-volume interaction among CNTs is denoted as the last term. The average excluded-volume contribution arising from rods with relative length *l* and $l^{\prime}$ could be presented as equation (3):


(3) \begin{eqnarray*}\rho ( {l,l{\mathrm{^{\prime}}}} ) = \frac{4}{\pi }\int | {u \times u{\mathrm{^{\prime}}}} |\big[ {\psi ( {l,u} )\psi ( {l{\mathrm{^{\prime}}},u{\mathrm{^{\prime}}}} )} \big]du{\mathrm{^{\prime}}}du.
\end{eqnarray*}

The maximum value for this term, 1, is achieved in the isotropic phase, while the locally ordered anisotropic nematic phase reduces the exclusion volume to a minimum due to the tubes’ ordered alignment. For the ideal Onsager rods model, the phase behavior is dictated by the confrontation between orientation entropy related-$\omega ( l )$ and translation entropy related-$\rho ( {l,\ {l^{\prime}}} )$ as a function of $c( l )$. While $\lambda ( {l,\ {l^{\prime}}} )$ is a specific term for the CNTs dispersion system that delineates the interactions between the tubes including attractive and repulsive forces. Hence, the theoretic fundament of the unique phase behavior of CNTs-superacid dispersion stems from the fourth term.

According to the aforementioned theory, the rigid-rod polymer resemblant CNTs can spontaneously form LC phase depending on their concentrations. Figure 3a–n schematically elaborates the dispersion state evolution of CNTs with their corresponding cryo-electron microscope (EM) images and birefringence patterns obtained in orthogonally polarized light. Concentration sections (*C*) expressed with the length *l* and the diameter *d* of rod particles are defined by Doi and Edwards [88,89]. At a dilute concentration ($C\ <\!\!\! < \ 1/{{l}^{3}}$), there is negligible interaction between CNTs established as shown in Fig. 3a and f, excepting long-range hydrodynamic forces. After dispersion reached the semi-dilute regime ($1/{{l}^{3}}\ < \ C\ < \ 1/( {d{{l}^{2}}} )$), as depicted in Fig. 3b and g, the rotational diffusion is restrained by the individual CNT steric hindrance. As the steric hindrance continues to dilate with increasing concentration, translation diffusion is also confined beside rotational diffusion (Fig. 3c and h), which the dispersion state is termed an isotropic regime ($C\ 1/( {d{{l}^{2}}} )$). As presented in Fig. 3k and l, when concentrations are within the three regions mentioned above, birefringence could not be observed *via* polarized optical microscopy (POM). The isotropic phase becomes unstable with some regions forming LC phases spontaneously when the whole system can receive additional entropy at the cost of reducing orientational entropy through upgrading translational entropy by unidirectional orientation. As demonstrated in Fig. 3d and i, this region achieves partial alignment, in which biphasic is employed to portray this coexistence of isotropic phase and LC phase. A distinctive birefringence motif of spindle-shaped LC droplets, or the so-called tactoids will be observed in nematic regions within the concentration range of biphase as shown in Fig. 3m. However, it's still challenging to discern the exact phase state of dispersion from the birefringence motif, due to the blurry boundary between the biphase and nematic phase.

Finally, the vanishing of the isotropic phase indicates the nascence of the nematic phase featuring special Schlieren textures (Fig. 3n), which present a long-range anisotropic orientation and homogeneous distribution (Fig. 3e and j). In addition, it is noteworthy that the phase transition concentration from isotropic to biphasic is closely related to aspect ratio (${{\varphi }_c} = 3.34d/l$) as predicted by the Onsager theory [85]. For instance, CNTs with varied aspect ratios demonstrate different LC phase transition points. This phenomenon can also be derived from viscosity-concentration profiles. The phase transition points and aspect ratios show a significant negative dependency (Fig. 3p). However, experimental values and theoretical numbers presents disparity, which stems from the poly-dispersity and counteraction between short-range repulsion and long-range attraction [58]. Figure 3q indicates that the fibers spun from the biphasic dispersion exhibit a lower specific strength and orientation factor than those from the nematic phase. The specific strength of the CNTFs derived from nematic wet-spinning dope exhibits a nearly constant value, which reconfirms the inheritance of CNTs local orientation arrangement from LC phase to CNTFs.

As for the CNTs dispersion, the prerequisite for forming a LC is the achievement of a stable dispersion of CNTs with a high concentration. First, the dispersants demonstrate a great influence on the homogeneity of spinning dopes. Comparing to amphiphilic compounds, superacids can endow CNTs with a mighty repulsive force due to the effective protonation process. Therefore, most currently reported LC dopes are based on superacids. Additionally, the instinct parameters of the CNTs will also affect their solubility. Besides, the pretreatment process serves as another contributing factor that directly affects the quality of the dispersion. It is reported that the introduction of trace amounts of oxygen *via* pre-oxidation can promote the protonation process of CNTs, thus improving dispersion [62].

However, the strong van der Waals force leads to a significant cohesive energy barrier, which may amount up to 186 mJ m^–2^ when approaching graphite cleavage energy [92,93]. Such a formidable energy barrier is challenging to overcome, which leads to inevitable CNTs self-aggregation. Heinz *et al*. [94] employed molecular dynamics (MD) simulations that include virtual **π** electrons showing more accurate interatomic potentials for graphitic materials with the solvents and polymer solutions, uncovering vigorous inclination of CNTs to revert back to the bundle state in common organic solvents. Therefore, the dispersion agents for CNTs ought to be deliberately selected. To date, three modalities are implemented to achieve high-concentration mono-dispersion of CNTs, which are attributed to their capabilities of screening or nullifying cohesive force between CNTs rest upon electrostatic repulsion, hydrophilic and hydrophobic interaction, and steric repulsion [95,96]. To endow the individual CNT with the aforementioned interactions, the CNTs need further modification including covalent functionalization [97,98], non-covalent adsorption [99–101], and charge transfer process [102,103]. There are many strategies to achieve covalent functionalization, including chemical oxidation [97], halogenation [104] and plasma activation [105], which is attributed to the fact that CNTs tend to be more spinnable when containing a higher level of functional groups.

However, it will lead to severely truncated length and defective *sp^2^* backbone of CNTs, which leads to inefficient load transfer in CNTFs and moderate conducting performance [106]. Thus, covalent functionalization is rarely utilized for preparing wet-spinning dope due to the egregious disruption of the CNTs intrinsic physical properties. Therefore, spinning dope preparation strategies are mainly discussed with the emphasis on non-covalent adsorption and charge transfer, including their physiochemical dispersion mechanisms and state-of-the-art exemplars.

### Non-covalent adsorption

**Figure 4.**
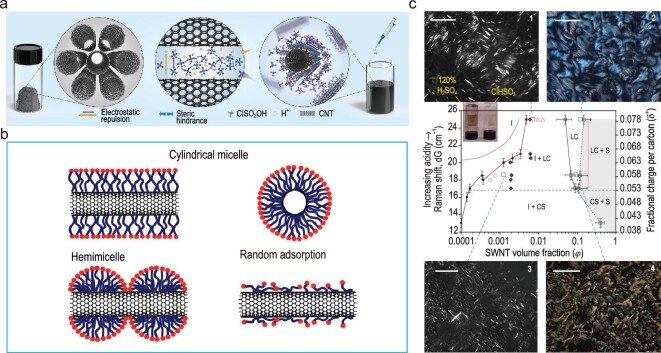
(a) Schematic illustration of the dispersion process of CNTs. (b) Three surfactants-CNTs supramolecular complex morphology. Adapted with permission from Ref. [108]. Copyright 2009 IOP Publishing. (c) Phase diagram of CNTs in superacids. (I) represents isotropic, (LC) represents LC, (CS) represents crystal solvate, and (S) represents solid. Digital pictures of 1.21 vol% CNTs in CSA (1), 12.1 vol% CNTs in CSA (2), 0.132 vol% CNTs in 120% H_2_SO_4_ (3), and 10.6 vol% CNTs in 120% H_2_SO_4_ (4) under polarized light. Scale bars: 50 mm (1) and 20 mm (2–4). Adapted with permission from Ref. [59]. Copyright 2009 Springer Nature.

Non-covalent adsorption is a non-destructive strategy for preparing wet-spinning dopes, where natural or synthetic amphiphiles have been identified as reversible adsorbents fixed to the sidewalls of CNTs. This could result in a sufficient electrostatic repulsion or steric hindrance to repel adjacent CNTs. Surfactants as an amphiphilic compound, uniting hydrophilic heads associated with solvents and hydrophobic tails adsorbed to CNTs [36,99,106,107]. Such surfactants-CNTs supramolecular complex is kinetically stable due to the virtue of electrostatic repulsion triggered from dissociated hydrophilic groups and steric hindrance provided by solvent-dissoluble groups around CNTs. As schematically illustrated in Fig. 4b, there are three broadly accepted surfactants-CNTs supramolecular complex models [108], (1) CNTs encapsulated as a cylindrical annulus [109], (2) surfactants adsorbed onto CNTs in a hemimicelle way [39], (3) surfactants randomly adsorbed around CNTs [110]. Sansom *et al.* [108] concluded that the morphology of the derived supramolecular complex was dictated by the surfactant species and surfactant/CNTs ratio. Recently, a research work focusing on LC behaviors of SWCNTs in an aqueous sodium cholate dispersion has unraveled the existence of sequential LC phase transition, which is consistent with the rigid-rod like polymers [68]. This phenomenon indicates that CNTs can be pre-oriented by the normal surfactant-assisted strategy, which is imperative for the fabrication of ideal CNTFs [18].

The macroscopic physical properties of CNTFs are tightly correlated with the length of the CNTs [37,38]. However, as a ubiquitously utilized and effectively operated approach to input energies in order to peel the individual CNT from bundle, ultrasonic oscillation could lead to other issues of cropping CNTs into short tubes, while simultaneously kinetically stabilizing surfactant-tube complex. It will severely impair the strength, electrical and thermal conductivity of the derived CNTFs [38,55,61,95,96,111]. Surfactant-tube affinity [61,112], pre-treatment [113] and different means of energy input [114,115] are major methods to alleviate the truncation effect aroused by sonication, which will be detailed in Supplementary note 1.

Although amphiphilic surfactants can be easily manipulated for CNTs dispersion by adsorption on the tube sidewalls, there are still certain drawbacks. (1) The ratio of CNTs/dispersant needs to be strictly controlled, because the excessive dispersant will raise a marked air-entraining effect thus deteriorating the CNTs dispersibility. Simultaneously, the osmotic pressure of excessive micelles will cause depletion-induced aggregation [54,116]. (2) A myriad amount of surfactant is required due to the large surface area of CNTs, while the residual dispersants inside the fibers serve as plasticizers. It could affect the CNTFs load transfer, thus significantly reducing their mechanical properties [113]. (3) Electrical and thermal conductivities of CNTFs are also dramatically impaired by the insulating residual dispersants. (4) Although a great deal of research has focused on cutting down or replacing ultrasonication processing, ultrasonic cavitation remains the most widely utilized, simple and effective approach to input energy for CNTs dispersion. As a result, the CNTs length is inevitably truncated, thus negatively impacting the CNTFs performance. These drawbacks lead to the deterioration of CNTFs performance, which can barely meet the demand of high-performance fibers. However, non-covalent surfactant adsorption provides an experimenter-friendly strategy for CNTFs preparation. Furthermore, the aqueous wet-spinning dispersion confers better compatibility of CNTs with other functional guest components for developing functionalized hybrid CNTFs [49–51,117,118].

### Charge transfer

Hitherto, CNTFs fabricated through non-covalent adsorption usually demonstrate unsatisfactory performance. Commencing in 2004, Smalley and Pasquali *et al*. [57] demonstrated the ability of superacids to spontaneously disperse CNTs. The formation of thermodynamically stable spinning dopes derived from the interaction between proton ionized from superacids and electron-rich CNTs. In this case, the protonation process takes place in CNTs dispersion, where a local charge polarization exists between the proton-CNTs complexes endowing CNTs with partial positive charges. Since the origin of CNTs dispersion stability is derived from the electron polarization between proton and CNTs, this process is defined as the charge transfer mechanism.

Subsequently, wet-spinning CNTFs based on superacids protonated dope have been extensively studied. These fibers have excellent full-scale properties based on their constituents’ large aspect ratio as protonation is reversible and non-destructive. In addition, owing to the sufficient unwarping of CNTs bundle and extremely high concentration of spinning dope, the individual CNT tends to form an LC domain, which contains micrometer size local ordering arrangement.

As schematically illustrated in Fig. 4a, the charge transfer mechanism was proposed [73] wherein the CNTs are reversibly protonated as polycarbocations along with corresponding counter anions. The individual dispersion of CNTs can be boiled down to the Columbic force and steric hindrance derived from a fractional positive charge on the sidewalls and congestion of conjugate base anions, respectively. The formation of polycarbocations can be interpretated as equation (4) [73]:


(4) \begin{eqnarray*}{{{\mathrm{C}}}_x} + y{\mathrm{HA}} \to \left[ {{{{\mathrm{C}}}_x}^{\delta + }{{{\mathrm{H}}}_y}^{\left( {1 - k\delta } \right) + }} \right] + y{{A}^ - },
\end{eqnarray*}

where *k* = *x*/*y* and δ is the fractional positive charge carried by each carbon atom. In fact, hydrogen evolution is implausible, while the almost constant I_D_/I_G_ from Raman spectrum during the CNTs dispersion in superacids corroborates the their intact *sp^2^* backbone. Hence, fractional electrons removal is more analogous to reversible polarization between CNTs and proton complex rather than a permanent electron transfer. This process can be understood in light of conformationally resemblant stiff-chain polymers dissolution behavior in superacids like poly(p-phenylene terephthalamide) (PPTA, i.e. Kevlar) in sulfuric acid or poly(benzobisoxazole) (PBO) in methanesulfonic acid. However, intact CNTs are devoid of nitrogen- or oxygen-containing groups which can promote protonation. Then, the formation and stability of the CNTs-proton complex is tightly intertwined with the stability of the conjugated base anion, i.e. A^–^. Therefore, the charge transfer mechanism triggered CNTs dispersion needs a very aggressive acid. This protonation protocol has been characterized both spectroscopically and microscopically, vindicating the depletion of *v*_1_ valence electrons from CNTs and orderly arrangement of superacid adjacent to CNTs. Relevant discussion is presented in Supplementary note 2.

As for the CNTs phase behavior in superacids, initial studies were conducted *via* rheologic and optical characterizations [102] (Supplementary note 3). In 2009, a mathematical model was established to depict the distinct CNTs phase behavior with polydisperse length and solvent-meditated attraction and repulsion [58]. For instance, the bio-phase emerges in poor solvents featuring thread-like structures composed of a stream of axially aligned but normally restricted SWCNTs. While in the same circumstance, solid phase is predicted by Flory's lattice theory [86] and Khokhlov's extension of Onsager’s theory [119]. Furthermore, broadening of the biphasic chimney is majorly attributed to the decrease of ${{\varphi }_c}$ rather than increase of ${{\varphi }_{nem}}$, which is reasonable for rigid-rod-like polymers according to the Onsager [85] or Flory [86] theories. The discrepancy between early theories and CNTs-superacid dispersion behavior stems from the fact that these theories focused only on ideal monodispersed Brownian rigid-rods with high aspect ratio in an athermal solvent interacting *via* mean field excluded-volume potential. Or it is assumed on the systems, which contain attractive interparticle interactions simply referring to the enthalpy driven effect of the mixture at a short range, while the phase behavior of CNTs in superacid is dominated by the counteraction between long-range van der Waals attraction and short-range electrostatic repulsion. Such attraction is weakly dependent on the acid strength, alternatively short-range electrostatic repulsion is strongly dependent on the acid strength. In tandem with the distinctive driving force, the CNTs polydispersity also wards off the application of earlier theories for CNTs dispersion. After incorporating these two deviations, an extension of Onsager's theory was developed. It forecasts that the isotropic-biphasic boundary approaches zero with decreasing acid strength to justify that isotropic and LC phases coexist at very low concentrations. Pasquali *et al*. [59] demonstrated a further study to analyze the LC phase formation mechanism. As presented in Fig. 4c, with more profound understanding of CNTs-solvent interactions, a phase diagram of SWCNTs has been summarized to depict the dispersion state of SWCNTs in varying acidity pragmatized through mixing of sulphuric acid and CSA. The experimental results were anastomosed to the theoretical values, which confirms that the CNTs-solvent interactions are indeed solvent-meditated in a short-range repulsion competing with a long-range attraction. The phase diagram reveals that a new crystal solvate phase occurred in poor solvents as long-range attraction pulling the rod together to yield a high-concentration phase, which consisted of aligned CNTs intercalated by a minimal acid.

By virtue of the elaborated phase diagram, it could offer guidelines for both wet-spinning dope and coagulation bath preparation. Primarily, a critical solvent acidity was defined (Raman shift, dG, ∼17 cm^−1^, corresponding to 100% H_2_SO_4_). To ensure the spinnability and compact structure of derived CNTFs, the wet-spinning dope should maintain LC feature with acidity higher than that of 100% H_2_SO_4_. Second, LC morphology is also dictated by the acidity. For instance, the CNTs dispersion with CSA (stronger acid, Fig. 4c2) demonstrates larger-size LC domains, lower defect density and more sleek domain orientation than those in 120% H_2_SO_4_ (Fig. 4c4). Meanwhile, a higher concentration also exhibits the analogous behavior comparing Fig. 4c1 to Fig. 4c2 and Fig. 4c3 to Fig. 4c4. At last, the proximity of isotropic concentration between CSA and athermal solvents from theoretical prediction [120] corroborates that van der Waals interactions triggered by CSA fully enabled it as the elusive athermal solvent for SWCNTs.

Since then, the investigation of wet-spinning CNTFs has focused on the CSA-CNTs dispersion system. According to an in-depth understanding of solvent-meditated inter-CNTs interactions and the mapping of the CNTs-super acid phase diagram, high-performance multifunctional CNTFs have been fabricated with CNTs-CSA wet-spinning dope, giving rise to a tensile strength level of 1 GPa.

In addition, the charge transfer mechanism not only contains superacid systems, but also includes alkali metal derived CNTs dispersion [103,121–123]. CNTs are also found to spontaneously dissolve in a series of aprotic organic solvents, including dimethyl sulfoxide (DMSO) and sulfolane with the appliance of alkali metals to form SWCNTs polyelectrolytes. However, the as-fabricated CNTFs by alkali metals can hardly be on par with those fibers spun from superacids owing to their rough surface morphology and loose internal structure, which only exhibit a tensile strength of 124 MPa and a Young's modulus of 14 GPa [70]. Meanwhile, cresol also demonstrates acceptable dispersibility [124,125]. It's still under debate whether the CNT dispersion state is monodispersed or dispersed in bundle state [126]. However, the cresol derived CNTs dispersion is still not spinnable.

In a nutshell, charges are transferred from superacids or alkali metals, respectively, to CNTs to form polycarboions for nullifying intertubular van der Waals forces through Columbic force. Specifically, an extension to the Onsager theory was developed to understand the superacid dispersion of CNTs with polydisperse length and solvent-meditated interactions. Benefiting from this theoretical model, a comprehensive phase diagram was portrayed to render the distinctive phase behaviors. The as-prepared spinning dopes are highly concentrated and liquid crystallined without any perturbation of *sp^2^* backbones and truncation of CNT length attributed to the non-destructive charging process. The CNTFs yielded in this way are highly oriented and densely packed, with distinguishing mechanical strength, electrical and thermal conductivities, representing the most promising way to produce high-performance CNTFs.

## FILAMENT SOLIDFICATION IN COAGULATION BATH

The coagulation process involves the transition of wet-spinning dope from a liquid or sol-gel state to a solid state. Diffusion controls the phase separation process, which is contingent on the liquid molecule diffusion kinetics and CNTFs solid phase transition thermodynamics. Therefore, it is critical to understand how the coagulation bath composition, coagulation rate, and temperature affect the coagulation process both kinetically and thermodynamically [37,41,44,127–129]. Ideally, appropriate coagulation bath modulation and suitable phase transition design is indispensable to obtain the ideal CNTFs [60]. This section mainly elucidates the coagulation mechanism in wet-spinning processes with a pivot on the impacts of the coagulation parameters on both macroscopic and microscopic morphology, aiming to provide insights for the pullulation of high-performance CNTFs from the coagulation mechanism.

### Coagulation mechanism

There has been well-established wet-spinning theoretical guidance in synthetic chemical fibers produced in the past few decades. For example, the dual diffusion process between solvent and coagulant in wet-spinning presents a significant influence on fibers’ morphology and porosity, while the phase transition is dominated by the kinetics and thermodynamics of the ternary component system throughout the wet-spinning process.

**Figure 5.**
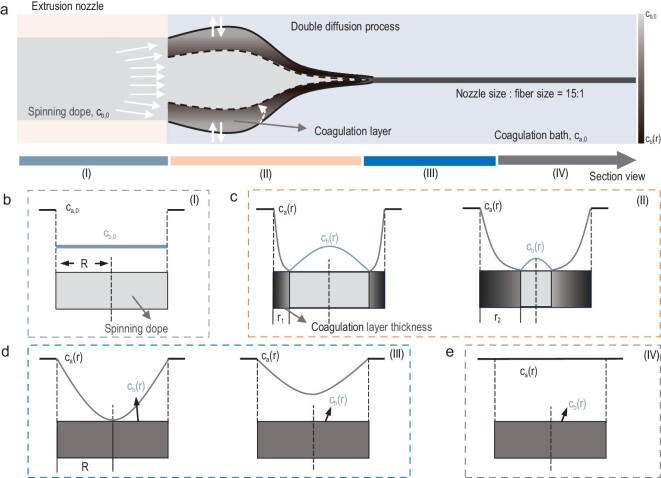
(a) General schematic diagram of the moving boundary of the CNTFs solidification-diffusion process. (b–e) The concentration evolution curves and corresponding solidification status for the coagulation stages Ⅰ, Ⅱ, Ⅲ and Ⅳ, respectively.

The diffusion rate between the solvent and coagulant is one of the decisive factors in determining the fiber morphology, compactness, porosity, and other physical properties (mechanical, electrical and thermal properties) [130]. The diffusion process is mainly contingent on the concentration difference between coagulation bath (${{C}_a}( r )$) and the spinning dope phase concentration (${{C}_b}( r )$). Thus, the concentration gradient at the dual interface is the main driving force to generate phase separation for fiber solidification. The solvent or coagulant diffusion behavior can be described by the classic Fick's law. As shown in Fig. 5a, the schematic diagram of CNTFs solidification-diffusion kinetic process ‘Moving Boundary’, including four stages Ⅰ, Ⅱ, Ⅲ, Ⅳ reveal the dynamic model of the concentration during fiber solidification in the dual diffusion process (Fig. 5b–e). The overall dispersion concentration variation has been indicated *via* gray scale changes in the schematics according to the color change trend scale bar (from light gray of ${{c}_{b,0}}$ to black of ${{c}_{b,( r )}}$), while the filament diameter changes from approximate nozzle diameter of 150 μm to final fiber diameter of 10 μm. The curves and corresponding rectangular gray color evolution region in Fig. 5b–e represent the cross-sectional solidification process for CNTs filaments at different coagulation stages. At stage I, the initial concentrations of spinning dope and coagulation bath are stabilized at ${{c}_{b,0}}$ and ${{c}_{a,0}}$, respectively. With the extrusion of dispersion from the spinneret, the dual diffusion and phase transition process take place at stage II, in which the solidified layer on the fiber surface gradually extends to the interior of the fiber and the solidified portion shows a concomitant increase. The solidification rate of wet-spinning could be estimated *via*  ${{r}^2}/4t$ [131], in which fiber solidification thickness (*r*) and solidification time (*t*) are the independent variables, while solidification rate is the dependent variable. The concentration gradient between the concentration of coagulation layer and solvent component is the major driving force for the dual diffusion. When solidification reaches stage III, the filament completes the full coagulation with an unchanged filament coagulant concentration (${{c}_{b( R )}}$), while the coagulation bath concentration outside the filament has not yet fully reached the diffusive equilibrium as the coagulant continuously diffuses to the outer layer. Eventually, the coagulant concentration boundary achieves final equilibrium at stage IV, in which both the coagulant concentration inside/outside of the fiber remain constant. Meanwhile, the mutual affinity between solvent and coagulant could lead to varied phase separation rates and thus different packing densities [82,132]. In the traditional wet-spinning process, a slow dual diffusion process is beneficial in obtaining densely packed fibers with a highly uniform and vacancy-free microstructure. However, the specific circumstances require analysis of various conditions to discern the relationship between solidification rate and the structure and properties of CNTFs. In addition, we also introduce the thermodynamics of the phase separation process that take place during the solidification process in Supplementary note 4.

In addition, there are still plenty of parameters, such as temperature, coagulant/solvent concentration, to affect the filament solidification process [133]. For instance, the higher wet-spinning temperature boosts affinity between solvent and coagulant, which could also lead to preferable fiber orientation accredited to the promoted fluidity and reduced dispersion viscosity and *vice versa*. In the low temperature and slow coagulation rate scenario, Sobhanipour *et al*. [134] demonstrated that polyacrylonitrile fibers usually present a non-circular cross-sectional morphology, such as beans or dog-bone shapes with restrained voids inside.

The coagulation process is the crucial step for determining the CNTFs morphology and macroscopic property. Although structural defects in fibers (e.g. micropores or localized disordered structures) are generally unavoidable, it is possible to modulate the morphological structure of fiber protofilaments by adjusting the concentration, temperature, and dual diffusion rate. In addition, the other parameters are conducive to optimize the macroscopic fiber configuration (cross-sectional morphology, porosity, core-sheath structure) and microscopic structure (CNTs orientation).

### Coagulation influences on CNTFs morphology and properties

Generally, the CNTs spinning dispersion prepared by different solvents (e.g. surfactants, protonic acid and some biomolecules) corresponds to different coagulation mechanisms, according to the specific precipitation mechanism for different solvent/non-solvent couples. In addition, the characteristics of CNTFs derived from superacid solvent or amphiphilic surfactant assisted dispersion demonstrate significantly different properties according to their distinctly varied solidification mechanisms.

In amphiphilic surfactant assisted dispersion system, various coagulants are used to achieve filament solidification. Vigolo *et al*. [36] prepared a 0.35 wt% CNTs wet-spinning dope with SDS assisted dispersion, which injected into a 5 wt% PVA coagulation for further filament solidification. However, due to the high molecular weight of 70 000, the PVA coagulant adsorbed onto CNTFs to form a boundary layer to further hinder the further diffusion between the solvent and the coagulant. As a result, the derived CNTFs form into a skin-core structure with porous microstructures, due to the interrupted dual diffusion process. Subsequently, Mukai *et al.* [55] found that sodium cholate (SC) surfactant coated on CNTs promoted the preparation of homogeneous CNTs dispersions. The diffusion of the solvent (water) into the coagulant isopropanol (IPA) resulted in a severe loss of surface activity of SC on CNTs. Meanwhile, the coagulant IPA diffused into the CNTs filament to exclude the solvent for solidifying the fiber *via* introducing compact van der Waals forces between CNTs. Afterwards, the resultant CNTFs were immersed in water for several hours to fully remove residual surfactant SC. Meanwhile, Jiang *et al.* [72] also utilized a similar solidification mechanism to further remove the solvent and surfactant with 600°C thermal annealing.

In addition, Razal *et al.* [56] systematically studied the coagulation bath effects, such as pH, crosslinking agents, or biomolecular precipitation solvent on CNTFs solidification process. When HA-CNTs dispersion was injected into controllable coagulants with varying pH values, the fabricated CNTFs generally exhibited a consistent flat morphology. In this work, injecting different dispersion into a calcium-ion coagulation bath could efficiently prevent the core-sheath structure formation. The calcium ion introduction into the coagulation bath could significantly accelerate the solidification rate, which could propel uniform surface structure formation with regular round shape. In addition, some common organic solvents have been also selected as the coagulant to optimize the solidification process. For instance, alcohol or acetone could effectively accelerate the dual diffusion process for fiber formation when applied as the solidification medium, which resulted in a thin and curly ribbon fiber configuration.

Strong acids, including fuming sulfuric acid, CSA, *etc.*, can enable the preparation of highly concentrated CNTs dispersions through the protonation triggered charge transfer effect. Spinning solution configured with fuming sulfuric acid is extruded into a coagulant ether, and the sulfuric acid rapidly flows out of the fibers to form a dense hard layer. When the ether evaporates, the fibers take on a collapsed structure (often referred to as ‘dog-bone’) [20]. Whereas, when the fibers are spun into dilute sulfuric acid or water, the fibers remain round and solidify in a more uniform and denser manner because of the restricted dual diffusion rate. Rapid solidification causes the fibers to preferentially form a solid surface. Due to capillary compression with evaporation of the coagulant, the fibers form a pleated structure. In 2009, 8.5 vol% CNTs dispersion could be prepared in CSA. Afterwards, the dispersion was spun in a 96% sulfuric acid coagulation bath for filament extrusion, but the viscous force at the interface in the coagulation bath leads to irregular fiber bending. It is proposed that this phenomenon can be avoided by using low viscosity coagulants, such as chloroform or methylene chloride. This also provides some guidance for the subsequent coagulant selection [59]. In 2015, Bucossi *et al.* [60] further investigated the effect of different coagulants (acetone, acetonitrile, chloroform, N, N-dimethylacetamide (DMA), deionized water, DMSO, ethanol, and n-hexane) on the fiber properties within the CNTs/CSA dispersion system. The varied coagulation baths composition could lead to the significant differences in the morphology and micro-structure of the derived CNTFs, which were mainly attributed to the CNTs van der Waals force, the diffusion rates, and interactions between the coagulation bath components and CSA. In such a scenario, these coagulants could be classified into several types with specific chemical reactions (acetone), vigorous reactions (deionized water, ethanol), or slow coagulation diffusion rates (chloroform, acetonitrile, DMA, DMSO). Figure S5a and b summarize the conductivity and tensile strength of CNTFs obtained with varied coagulation bath compositions. When acetone is utilized as the coagulation bath, the thus-derived CNTFs tend to demonstrate optimal tensile strength along with electronic/thermal conductivities.

In addition, Kim *et al.* [41] analyzed the effect of acetone as a coagulant on the macrostructure and microstructure of CNTFs. The mass transfer rate between the solvent and non-solvent has been utilized to control the CNTFs morphology. When the wet-spinning is conducted with a small diameter spinneret under the consistent mass transfer rate, the accelerated solidification rate leads to a limited radial concentration gradient within the CNTFs. Figure S5c illustrates the morphological structural changes of the fibers during the coagulation process. Herein, we define the roundness (shape factor) of the fiber cross-section as equation (5):


(5) \begin{eqnarray*}
S{{F}_R}\ = {\mathrm{\ }}\left( {4{\mathrm{\pi A}}} \right)/{{P}^{2}},
\end{eqnarray*}

where *A* is the cross-sectional area of the fiber, and *P* is the fiber perimeter. During solidification, *P* may be fixed, whereas *A* decreases with further solidification. The $S{{F}_R}$ becomes <1, which is the case for non-circular cross sections. In addition, it is possible to reduce the radial concentration gradient by reducing the diameter of spinneret to obtain an approximately circular cross-section morphology with an $S{{F}_R}$ close to 1. Figure S5d and e show cross-sections of CNTFs with $S{{F}_{R}} \ 1.0 $ and $S{{F}_{R}} < 1.0 $. The results show that controlling the concentration gradient can obtain CNTFs with circular cross-sectional morphology.

Upon examining these analytical outcomes, we have engaged in a sophisticated evaluation regarding the association between the fiber properties of CNTFs and their corresponding coagulation conditions within surfactant-enriched versus strong acid-based systems. The coagulation mechanism observed under the surfactant system predominantly unfolds as a slow bidirectional diffusion process. In stark contrast, coagulation within the strong acid system not only proceeds through a more accelerated bidirectional diffusion but is also enhanced by an intrinsic chemical reaction. The implications of these studies suggest a critical dependency of the mechanical and physical attributes of CNTFs on the rapidity of the coagulation rate. Thus, these empirical insights could guide the development of a conjectural yet economically feasible and practical process for fabricating high-performance fibers through orchestration of the rapid coagulation rates. This would encompass the methodical design of chemical reactions among different constituents, as well as the modulation of reaction kinetics and coagulation bath densities.

## POST-TREATMENT

In addition to the CNTs wet-spinning dope preparation and coagulation solidification process, the post-treatments such as multi-stage drafting and thermal annealing are indispensable for further promoting the alignment and crystallinity with desirable fiber structures to achieve optimal physicochemical performance.

### Drafting

**Figure 6.**
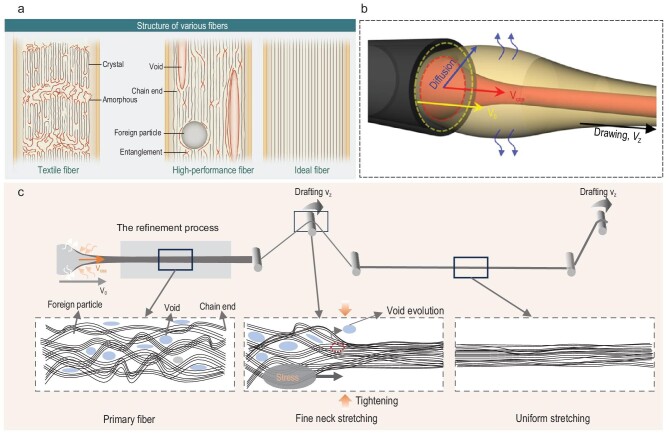
(a) Schematic structures of various fibers, (Left) typical commodity textile fiber, (Middle) high-performance fibers, (Right) ideal fibers. Adapted with permission from Ref. [18]. Copyright 2008 American Association for the Advancement of Science. (b) Schematic illustration of linear velocity at spinneret determined by coagulation effect. Adapted with permission from Ref. [41]. Copyright 2022 Elsevier. (c) Schematic illustration of the fiber structure evolution during the drafting process, such as random arrangement, fine neck stretching and uniformly oriented structure.

Figure 6a schematically illustrates the structure difference among conventional fiber, high-performance fiber and ideal fiber [18]. The conventional textiles or high-performance fibers contain a complex configuration, with a combination of amorphous, crystalline regions as well as voids, foreign particles, tangles and defects. In comparison, the ideal fiber presents all aligned long-chain molecular segments or structural units, without any defects. Indeed, some defects can be partially eliminated by the continuous drafting, rendering the defective nascent fiber into a densely-packed and highly-aligned structure. Thus, it is necessary to systematically understand the fiber microstructure evolution as well as the corresponding properties’ advance during the drafting process. Draw ratio is an important parameter for the wet-spinning process. Theoretically, draw ratio is calculated *via* the following equation (6) [41,44]:


(6) \begin{eqnarray*}
{{D}_R} = {{v}_z}/{{v}_0},
\end{eqnarray*}

where ${{v}_z}$, ${{v}_0}$ are the winding speed and the wet-spinning dope extrusion speed, respectively. In general, ${{v}_0}$ is determined by the flow rate of the spinneret (*Q*) and the aperture cross-sectional area of the spinneret (*A*). In addition, draw ratio is also determined by the wet-spinning fluid kinematics, which includes the shear effect that causes a distribution of velocity gradients in the spinning liquid, as well as friction effects from the spinneret side wall that determine the extrusion velocity. As schematically illustrated in Fig. 6b, the actual extrusion velocity (${{v}_{coa}}$) of the spinneret is corrected by incorporating the coagulation effect, and the actual draft ratio [41], which is mathematically expressed as equation (7):


(7) \begin{eqnarray*}
D_R^{\mathrm{*}} = {{v}_z}/{{v}_{coa}}.
\end{eqnarray*}

As demonstrated in Fig. 6c, the CNTFs structural evolution during the drafting process, including the CNTs orientation and dense packing after the extrusion swelling at the spinneret, leads to fine-necked stretching and multi-state drafting during the roll drafting process. The primary fiber obtained *via* the coagulant solidification will experience multi-stage CNTs stretching, disentangling and aligning under a high stress state. The defects will fuse together, shrink, and even vanish during the multi-stage drafting treatment. For some traditional wet-spinning fibers, negative draw ratios are necessary during the solidification process to eschew the fracture of primary fibers. Generally, the solidification process of CNTFs in the superacid system completes in a short time. As shown in Fig. 6c, when the nascent gel-state filament passes through the roll axis, it will be subjected to stress to form the fine necking. With the increase of the drafting ratio of the primary fiber, the stress will cause the molecular chain segments that have not been fully oriented to begin to stretch. The molecular chain segments are uniformly drafted to achieve a highly oriented fiber structure [135–137]. The detail Hyper-Spin-Line (HSL) model and the drafting process for enhancing CNTFs have been illustrated in Supplementary note 5.

### Heat treatment

The heat treatment is a necessary procedure to achieve highly crystalline domains along the axial direction of the fibers, which in most cases increases strength and modulus [138]. CFs with lamellar graphitic microcrystals stacked in the axial direction usually display light weight, high strength, high modulus and great thermal conductivity, which demonstrate broad application potential in aerospace craft and missile systems [139–143]. Simultaneously, CNTFs could significantly enhance the inter-tubular interaction, even forming covalent bonds after thermal annealing treatment. The thermal treatment could be generally divided into four stages: heat-setting, pre-oxidation, carbonization and graphitization [42,83]. In Supplementary note 6, we present the typical CF heat treatment process along with the structural evolution.

However, the current tensile strength of CNTFs only achieve 10% of the theoretical strength of an individual CNT. The mechanical strength of CNTFs is mainly limited by the shear strength between CNTs while the contact area between neighboring nanotubes is proportional to the shear strength. The contact area depends on the degree of polygonization or CNTs collapse [144–146]. It is well-known that the strong interactions between the layers during the preparation of CFs are generated at the graphitization stage, forming a ribbon-like stacking structure. Theoretically, the flattened structure of collapsed CNTs can also improve the intertube shear strength. According to the Monte Carlo model, SWCNTs bundle can be rearranged into multi-walled CNTs (MWCNTs) or more complex graphitic nanoribbons by agglomeration under high-pressure or high-temperature conditions [147].

**Figure 7.**
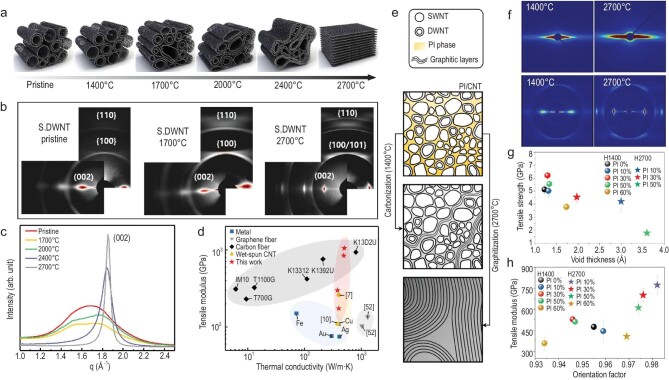
(a) Schematic illustration of CNTs coalescence *via* annealing temperature. (b) 2D wide-angle X-ray scattering (WAXS) patterns of the structure evolution of CNTFs after annealing. (c) Equatorial radial profiles showing the transition from bundle to highly crystalline graphitic domains upon high-temperature annealing. (d) Comparison of thermal conductivity as a function of tensile modulus with other CFs, CNTFs, and graphene fibers (References from the original article). Adapted with permission from Ref. [42]. Copyright 2022 American Association for the Advancement of Science. (e) Schematic illustration of the cross-section of PI-CNTFs as a function of the heat-treatment temperature. (f) 2D small-angle X-ray scattering (SAXS) and WAXS patterns of PI-CNTFs with 30% PI heat treated at 1400°C and 2700°C. (g) Relationship between tensile strength and void thickness. (h) Relationship between tensile modulus and orientation factor of (002) plane. Adapted with permission from Ref. [83]. Copyright 2022 Elsevier.

At present, most of the experimental studies need to be combined with MD simulations in order to understand CNTs bundle structure evolution during the heat treatment. CNTFs are usually put into a vacuum oven at 170°C for drying treatment over 24 h to eliminate the internal stress [37]. To avoid the intrinsic defects induced by structural damage, some polymers (such as polyarylamide, polyamido acid, *etc.*) have been introduced to restore the covalent connection between CNTs *via* carbonization processes. Lee *et al.* [42] explored the carbonization and graphitization of wet-spinning CNTFs with highly aligned SWCNTs or MWCNTs bundle, which induced the nanotubes to collapse and form a multi-interior walled structure. The nanostructures formed a network of tightly packed graphitic domains connected to each other, as shown in Fig. 7a. During the carbonization process, defects such as ends, dangling bonds and Stone-Wales of CNTs will act as active sites, leading to the reconstruction of the CNTs interval into covalent bonds [147]. Subsequently, a compression mechanism polymerization process occurs with increasing temperature, and finally graphitized grain growth and orientation planarization are completed after annealing at 2700°C. The thermal evolution process may be nanotube collapse driven by van der Waals forces. Whereas vacancies induce carbon structure fusion through a zipper-like mechanism that allows atoms to reorganize successively on the individual tube lattices of neighboring nanotubes. Simultaneously, other topological defects could also lead to CNTs fusion. In this process, the MD simulation confirms that SWCNTs bundle agglomerates to form MWCNTs, which is identified as a single-walled and multi-walled transition of the ‘mending-tearing’ mechanism [146].

After graphitization treatment, Fig. 7b and c’s analysis of WAXS results presents that carbon-CNTs fibers (C-CNTFs) can eventually achieve highly crystalline regions, while the high thermal conductivity is mainly attributed to the large crystallite length (L_a_) content and the corresponding reduction of phonon scattering. Meanwhile, the reduction of (002) lattice spacing enhances the interlayer interaction force. Figure 7d summarizes the tensile modulus and thermal conductivity comparison of C-CNTFs with other metal wires, CFs, graphene fibers, and other wet-spinning CNTFs. C-CNTFs already outperform most CFs, achieving the highest thermal conductivity of 496 ± 6 W m^–1^ K^–1^, the highest modulus of 1049 ± 105 GPa, and the highest strength of 7 GPa. Subsequently, to enhance the tube-to-tube interaction force, polyimide (PI) molecules were introduced into CNTFs [83]. The CNTs bundle structure evolution has been schematically illustrated in Fig. 7e, where the polymer molecules firstly transform into a graphite-like layer. As shown in Fig. 7f, the effects of the structural evolution of the fibers produced at different heat treatment temperatures on the fiber properties were investigated through SAXS and WAXS, which indicates that the C-CNTFs obtained by heat treatment at 2700°C contains large pores. However, the increase in the void thickness caused by the folding, splitting, or tilting of the graphite layers leads to tensile strength deterioration (Fig. 7g).

In brief, the CNTFs heat treatment is the key for preparing ultra-strong, ultra-high modulus, and ultra-high thermal conductivity fibers, mainly through the thermal evolution of the fiber structure to achieve the growth of the crystal size and enhancement of the interlayer interaction force.

## STRUCTURE AND PROPERTY OF CNTFS

In contrast to the wet-spinning strategy, there are two other representative spinning methods, such as array spinning from drawable CNTs vertical array [27–30] and direct dry spinning from FCCVD [31–34] to produce CNTFs. CNTs vertical array spinning involves the continuous drawing of CNTFs from vertically aligned CNTs arrays, wherein the individual CNT bundles are twisted to form fibers. In such process, numerous defect structures are generated inside the derived CNTFs, including amorphous carbon, residual catalyst, and the consequent porosity, which severely deteriorates the mechanical and other features of derived CNTFs. Besides, the stringent environmental requirements of the fabrication process and the high production costs limit large-scale industrial manufacturing. On the contrary, direct dry spinning from FCCVD offers a lower production cost but still suffers from challenges in controllable growth and mass production of CNTFs. In contrast, the wet-spinning strategy is the most promising strategy to produce CNTFs at a large scale, according to the massive treatment of CNTs dispersion and industrialized wet-spinning technique.

Hence, there is a critical need to address the pressing challenge of further optimizing the wet-spinning process to yield high performance CNTFs. This chapter will commence with an analysis of the intrinsic structure and properties of CNTs raw materials, followed by a discussion on the structure-performance relationship of CNTFs. Additionally, it will provide a summary of the strategies developed in recent years to augment fiber performance.

### Mechanical strength

#### Theoretical considerations of CNTFs

**Figure 8.**
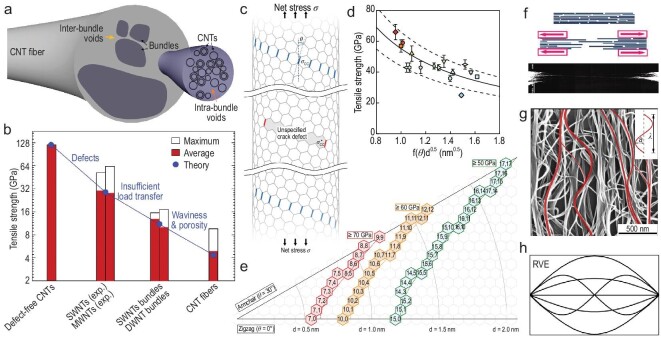
(a) Hierarchical perplexing microstructures of CNTFs. Adapted with permission from Ref. [41]. Copyright 2022 Elsevier. (b) Breakdown of assembly tensile strength at the nanotube, bundle, and fiber levels from defect-free CNTs. Adapted with permission from Ref. [136]. Copyright 2018 Elsevier. (c) Schematic illustration of CNTs breakage at the defect edge when stress is applied. (d) Tensile strength plotted as a function of $f( \theta ){{d}^{ - 0.5}}$. (e) Empirical contour map indicating the influence of diameter and chirality on tube’s strength. Adapted with permission from Ref. [154]. Copyright 2019 Springer Nature. (f) Schematic illustration of intertube sliding failure mechanism and SEM micrograph of actual fractography of CNTFs. Adapted with permission from Ref. [155]. Copyright 2011 American Chemical Society. Actual SEM images (g and h) and the representative volume element (RVE) of the wavy CNTs bundle in the CNTs networks. Adapted with permission from Ref. [136]. Copyright 2018 Elsevier.

Despite the extremely high tensile strength of 120 GPa for an individual CNT originated from the robust *sp^2^* hexagonal carbon lattice backbone, the strength of the vintage performing CNTs macro-assemblies are still inferior to 10 GPa. The undoing of this property inheritance from nanotubes to fibers can be attributed to the fibers’ hierarchical microstructures as illustrated in Fig. 8a [41]. It spans over multiple lengths scale through forming a bundle of submicron-scale from nanoscale individual CNT and then forming microscale fibers from wavy and entangled bundles. To clinch this matter, some theoretical models [148,149] have been proposed to quantitatively delineate the mechanical degradation mechanisms of CNTFs with multi-scale microstructures. For instance, Gao *et al.* [136] developed a model to quantify and paraphrase the breakdown of material strength at the nanotube, bundle, and fiber levels as presented in Fig. 8b. The intrinsic defects situated in an individual CNT results in a typical 24.3% strength reduction from 120 GPa to 29.2 GPa. Meanwhile, the insufficient load transfer in closely-packed bundles further leads to a reducing estimation of 37.5% and a derived strength of 11.0 GPa. Ultimately, the mismatching engagement and imperfect alignments between CNT bundles bring about 38.5% tensile strength reduction to 4.2 GPa, which is comparable to the values for CNTFs reported at that time. Along this line, we will discuss the three scaling levels of CNTFs, i.e. individual CNT, bundle, and macroscopic fibers, with a focus on the most cardinal ingredient at each level to further render the mechanical reinforcement mechanism.

The basic lattice structure of the individual CNT is crucial for determining the final derived CNTFs. However, defects, such as atomic vacancies [150], topological [151], or helical structural [152] or Stone-Wales defects [153] are inevitable within long-range CNTs. Two main factors that determine the CNTs tensile strength are the CNTs length and the defect properties, including defect density and detailed microstructures. These defects can illuminate the gulf between the low pragmatic strength of micrometer-long SWCNTs (30 GPa on average) and the high predicted value for defect-free CNTs [154]. Concretely, for a continuously increased SWCNT length from 0.01 to 10 mm, a continuous reducing trend in tensile strength was detected from 138 to 56.1 GPa and 108 to 49.5 GPa with the presence of 2% topological defects and 1.5% vacancy defects, respectively. It indicates that the tensile strength loss in the primary level is mainly attributed to the CNTs defects with a distinct length dependency. Nevertheless, the intrinsic strength of CNTs is multivariate-dominated, which is also affected by their intrinsic features, such as chiral structure and diameter. Takakura *et al*. [154] demonstrates small-diameter, near-armchair CNTs exhibiting the highest tensile strengths up to 66 GPa *via* a microelectromechanical system device. The angle $\theta $ between the stress applied to the axial direction of the CNTs and the C−C bond directions in tandem with the concept of stress concentration has been put into correlation, which stems from classical linear elastic fracture mechanics. As schematically illustrated in Fig. 8c, effective stress applied on the C−C bonds, ${{\sigma }_{cc}}$, is determined by the net stress $\sigma $ and $cos\theta $, while the defect edges withstand the concentrated stress. ${{\sigma }^*_{cc}}$ corresponds to classical linear elastic fracture mechanics. In this case, the brittle fracture occurs once a C−C bond in the weakest defect crack breaks. First, factor in chiral structure, as the chemical bonds feel different effective stress even under the same net stress. ${{\sigma }_{cc}}$ can be denoted as ${{\sigma }_{cc}} = f( \theta )\sigma $, where $f( \theta ) = \frac{1}{2}[ {( {1 - v} ) + ( {1 + v} )\cos 2\theta } ]$ and *v* is the Poisson ratio. Furthermore, albeit the size and type of defects are difficult to express quantitatively, a CNTs diameter-related parameter was defined to describe the effect of stress concentration, $\varphi ( d )$ that scaling with ${{d}^a}$. Then the concentrated stress can be expressed as ${{\sigma }^*_{cc}} = \ \varphi ( d ){\mathrm{\ }}f( \theta )\sigma $. Since the σ*_CC_ is a constant value for bond fracture, an empirical formula regarding the failure tensile strength ${{\sigma }_f}$ is obtained as equation (8) [154]:


(8) \begin{eqnarray*}
{{\sigma }_f} = Cf{{\left( \theta \right)}^{ - 1}}{{d}^{ - a}},
\end{eqnarray*}

where tensile strength can be plotted as a function of $f( \theta ){{d}^{ - 0.5}}{\mathrm{\ }}$shown in Fig. 8d, *C* and *α* are empirical constants. They were fitted to the experimental data to obtain $C\ = \ $$55$ GPa nm^0.5^, $a\ = \ 0.5$. In conjunction with these two factors, chiral structure and diameter size, a contour map in Fig. 8e is given to delineate the diameter size and chiral index at the 70, 60 and 50 GPa critical strengths, which is known as the minimum threshold requirement for constructing a space elevator [154].

In brief, the strength loss of CNTs is inevitable due to the existence of intrinsic defects. Meanwhile, the defects impairing the CNTs strength is regulated by multidimensional variables, including their length, diameter, chirality, defect types and densities. Within a given defect density and type, shorter lengths, smaller diameters and armchair chiral structures are favorable for preserving the intrinsic ultrahigh strength of flawless CNTs [136].

By virtue of the strong cohesive binding energy, localized densely packed bundles are formed during the managing of individual CNTs into macroscopic assemblies, which is the secondary structure unit in the CNTFs. There are two failure mechanisms for bundles, including CNTs backbone breakage and intertube sliding. These two mechanisms demonstrate a rate-dependent failure phenomenon, in which high rates induce cascade-like breaking, meanwhile intertube sliding occurs at low rates [156,157]. Suekane *et al.* [158] investigated the inter-nanotube static friction for highly crystalline CNTs. A low inter-nanotube static friction force of ∼0.43 nN and a stress of 2 MPa is revealed. Such marginal interfacial shear strength makes ${{l}_c}$ pragmatically unattainable thus leaving the failure mechanism at intertube sliding. In the scenario of wet-spinning CNTFs, the pre-treated CNTs are highly crystalline and the intertubular shear strength is more approximate to the lower boundary value. Fractography of CNTFs fracture surface shown in Fig. 8f in tandem with *in-situ* Raman spectroscopy further corroborate the intertube sliding failure mechanism [159]. Hence, the strategy of increasing the interfacial shear strength is more promising. A well-received theoretical model with simplicity is proposed by Vilatela *et al.* to analyze the intertube sliding failure mechanism as described in equation (9).


(9) \begin{eqnarray*}
{{\sigma }_2} = \frac{1}{6}{{\Omega }_1}{{\Omega }_2}{{\tau }_{\mathrm{F}}}L,
\end{eqnarray*}

where, *L* is the mean length of the element, ${{\Omega }_1}$ is the ratio between the outermost graphene lamellae and total number of walls expressed as $1/N$,*N*, ${{\Omega }_2}$ is the fraction of the surface of the outer graphene wall of the element in contact with neighboring elements, indicating the deformability of elementary CNTs. Based on classical elasticity theory, the CNTs cross-section area, *a*, the equivalent perfectly round tube radius *R*, a factor $\alpha $ related to the strength of bonding between layers $( {1 \le \alpha \le 3} )$, flexural rigidity of graphene layer *E* and surface energy $\gamma $ are incorporated to calculate the value of ${{\Omega }_2}$ as equation (10):


(10) \begin{eqnarray*}
{{\Omega }_2} = \frac{{6a}}{{2\pi R}} = 1 - \frac{1}{R}\sqrt {\frac{{{{N}^\alpha }E}}{{2\gamma }}}.
\end{eqnarray*}

Two key parameters, contact area (${{\Omega }_1}{{\Omega }_2}L$) and interfacial shear strength $( {{{\tau }_{\mathrm{F}}}} )$, can be gleaned from the as-mentioned model. First, regarding contact area, extra value could be realized *via* assembling CNTs with fewer walls, larger tube diameters and longer lengths. In addition, polygonization referring to flattening of tubes will lead to an increase of ${{\Omega }_2}$ attributed to the step-up of bonding area. When CNTs flatten to a certain extent, they form collapsed structures, as seen in larger diameter CNTs with thin walls [160]. Hence, CNTs with collapsed structures are deemed to exhibit higher ${{\Omega }_2}$ value with a higher tensile strength for each bundle. This conjecture has already been confirmed through MD simulation by Zhang *et al.* [161]. It indicates that while the critical pressure is applied, the CNTs exhibited a collapsed state, which still maintained the collapsed structure at atmospheric pressure after the pressure was withdrawn. The intertube friction was enhanced by a factor of 1.5–4 contingent on tube chirality and radius. Moreover, collapsed tube structures will also experience extended pairwise Lennard-Jones interactions, which could provide an advantage for stacking dislocation over regular and dislocation dipole stacking [135]. Under the above-mentioned instructions and theoretical models, recently, a breakthrough of wet-spinning CNTFs has been pragmatized *via* thermal treatment to increase the interactions between graphitic layers through CNTs collapse, coalescence, and ultimately graphitization. Second, in a bid to eliminate intertube sliding, crosslinking was established within each bundle *via* utilizing moderate electron-beam irradiation. Then, a 30-fold increase of the bending modulus was achieved [162]. This mechanical reinforcement strategy *via* interfacial modulation will be discussed in the following subsection.

Moving into the tertiary level, CNTFs feature wavy and interlocked bundles as shown in Fig. 8g. It is feasible to envisage that the fiber structure resembles that of a bundle while substituting the subunits from tube to bundle but with surging perplexity. In such a scenario, the bundle length is impossible to detect since no distinct termination is observed for bundles. Furthermore, the bundle tends to form a curly topology along its profile attributed to its large aspect ratio and omittable bending resistance. The intricate wavy topology can be extracted from SEM in Fig. 8g to give a simplified representative volume element (RVE), modeled by CNT bundles with a non-uniform distribution of waviness as illustrated in Fig. 8h [136]. Based on its relatively loose and wavy structural features, we can derive two mechanically crucial factors from a broad landscape, the degree of CNTFs compactness and orientation. In fact, the compactness and orientation are intertwined to all intents to a large extent, in which the improvement in compactness is proportional to the increase in alignment. Tremendous works have been put forward to vindicate the strong correlation between these two factors to promote fiber strength. Simply dilating the draw ratio at the filament greatly boosts the internal orientation of the CNTFs alongside the promotion of intimate contact between the bundle to inhibit void formation with a denser overall structure.

The tactics for fabricating highly dense packing and aligned wet-spinning CNTFs have already been covered in the aforementioned sections. In Supplementary note 7, we further elaborate on the introduction of a second mediator during the wet-spinning process to inhibit the intertube slip failure mechanism, thereby strengthening the interactions between tubes and thus producing high-performance CNTFs.

### Electrical properties

Highly conductive wires with ultra-light features are highly desirable for aerospace and large-scale smart grid applications. By virtue of the excellent intrinsic conductivity property of CNTs, CNTFs have been considered as the promising candidate for the next generation of conducting wires based on their excellent flexibility, high capacitance, great electrochemical stability and ultra-light weight [163].

#### Electronic conductivity mechanism of CNTFs

**Figure 9.**
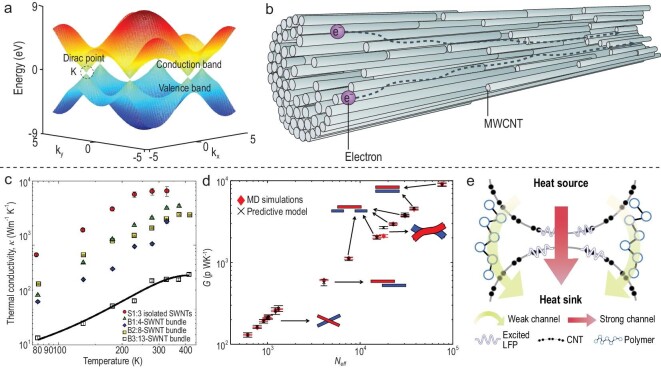
(a) Electronic structure of graphene calculated within a tight-binding model consisting only of π electrons. Adapted with permission from Ref. [164]. Copyright 2006 IOP Publishing. (b) A model of electron hopping within a conducting fiber made from aligned MWCNTs. Adapted with permission from Ref. [169]. Copyright 2017 Springer Nature. (c) Variation of thermal conductivity of SWCNT bundle with different size. Adapted with permission from Ref. [170]. Copyright 2018 AIP Publishing. (d) Intertube heat conductance as a function of the total ‘effective’ number of interatomic intertube interactions, obtained in MD simulations and predicted by a mesoscopic model. Adapted with permission from Ref. [171]. Copyright 2016 American Physical Society. (e) Schematic of polymer-broadened heat transport channels for contacted CNTs. Adapted with permission from Ref. [172]. Copyright 2022 American Physical Society.

CNTs have a unique electronic energy band structure that endows them with excellent electrical properties. The electronic energy band structure of CNTs can be analyzed from two-dimensional graphene, as shown in Fig. 9a [164]. In the two-dimensional inverse space of X and Y, there are six points that coincide with the Fermi surface, i.e. the energy is 0, referring to ‘Dirac point’. The band gap (${{{\mathrm{E}}}_{\mathrm{g}}}$) of CNTs can be regulated by their chirality, which categorized them into metallic and semiconducting. The conduction band and valence band do not contain any overlap near the Fermi surface. The band gap is predicted to be ${{E}_{g}} \approx 0.7{\mathrm{\ eV}}/{\mathrm{dt\ }}( {{\mathrm{nm}}} ) $ [165]. Theoretically, semiconducting CNTs have a high mobility 10^5^ cm^2^ V^–1^ s^–1^ as well as a current-switching ratio of 10^6^. Thus, it has a high potential for application in the field of transistors [166]. Meanwhile, the metallic CNTs are the ideal conductors to achieve high conductivity, in which the band gap between conduction band and valence band is approaching 0 without considering the effect of curvature, strain, *etc*. There exists an electronic density of states where the theoretical current density is 10^9^ A cm^–2^, which is three orders of magnitude higher than that of conventional metal of 10^6^ A cm^–2^. The current CNTs electrical properties exhibited in the Latinger liquid, display the ballistic transport behavior of quantum transport [167]. The quantum conductance in the ballistic transport state is $4{{e}^2}{{h}^{ - 1}}$ (*e* and *h* are the elementary charge and Planck's constant, respectively) without impurities as well as scattering. In fact, the real CNTs contain plenty of lattice defects, impurities, *etc.*, which deteriorate the CNTFs electrical conductivity [168]. As shown in Fig. 9b, the electrical properties of the macroscopic assemblies of CNTFs can be understood in terms of the 3D variable range hopping (VRH) model [169]. It is commonly understood that charge can jump rapidly from the external CNTs to the internal CNTs, and then transport efficiently along the axial direction of the fiber. In contrast, electron-leap conduction is the quantum mechanical tunneling of electron-assisted phonons from one domain state to another. The conductivity enhancement is limited by the intrinsic defects of CNTs (vacancies, *sp^3^* hybridization, *etc.*) as well as CNTs interfacial resistance. In Supplementary note 8, we further elaborate the strategy of enhancing electrical conductivity by introducing a secondary medium to optimize fiber density and alignment. Additionally, we draw upon other post-treatment processes, including doping, electroplating, CVD, and magnetron sputtering, to provide guidance for preparing highly conductive fibers *via* wet-spinning.

### Thermal properties

Thermal interfacial materials with light weight and high thermal conductivity properties are greatly in demand for aerospace heat sinks, electronics, batteries and other thermal management applications [140,142].

#### Thermal conductivity mechanism of CNTFs

MD simulations reveal that CNTs have a high mean phonon free range with ballistic transport features [173,174]. Thus, the CNTs usually exhibit a very high theoretical value, up to 3000–6600 W m^–1^ K^–1^ [175]. Experimentally, the thermal conductivity of SWCNTs could achieve about 3500 W m^–1^ K^–1^ [24], while MWCNTs were characterized to be about 3000 W m^–1^ K^–1^ at room temperature [176].

Theoretical calculations demonstrate that CNTs thermal transport is mainly contributed to by phonons, i.e. the vibrational heat transfer of the lattice [177]. In general, the thermal conductivity of CNTs can be expressed as equation (11):


(11) \begin{eqnarray*}
{{\kappa }_{ph}} = \mathop \sum \limits_i \frac{1}{3}{{c}_i}{{v}_i}{{l}_i},
\end{eqnarray*}

where ${{c}_i},{{v}_i}$ and ${{l}_i}$ are the unit volume heat capacity, velocity, and mean free path (MFP) of the *i-*th phonon mode, respectively. The variation of the average phonon mean free path is one of the key factors to determine the thermal conductivity of CNTs. Phonons are quantized forms of lattice vibrations, which determine the single-phonon thermal conductivity [178]. For SWCNTs, the phonon mean free path is not only influenced by low-frequency modes but also by factors such as CNTs length, defect density, and crystal structure. The finite size of CNTs restricts the motion of phonons, resulting in a size-dependent thermal conductivity *via* ballistic transport. MD simulations explain that when the phonon mean free path in CNTs is <40 nm, it will present ballistic transport without phonon scattering or other effects [179,180]. However, phonon scattering takes place when the phonon mean free path exceeds 40 nm. Then it eventually transits to fully diffusive transport as the CNTs length increase, with a decrease in thermal conductivity. Along with the growth in crystal size, the thermal conductivity of CNTFs will increase accompanied by the extended average phonon mean free path. Che *et al.* [175] suggest that an increase in vacancies and defects in CNTs significantly reduces their thermal conductivity. This reduction is attributed to the presence of vacancies and defects causing increased phonon scattering, thereby decreasing the average phonon mean free path.

Subsequently, researchers carried out MD simulations on the intertube heat transfer between CNTs bundles, which is essentially the heat transfer of the CNTFs secondary structure. As shown in Fig. 9c [170], compared to a single SWCNT, the thermal conduction of SWCNTs bundles is significantly hindered due to intertube interface thermal resistance, which causes phonon scattering, leading to a reduced thermal conductivity [181]. According to kinetic theory, the reason for the logarithmic decay of thermal conductivity with increasing bundle size is ascribed to the increased phonon scattering rate between adjacent SWCNTs. The interfacial resistance between CNTs has a significant correlation with the contact area and density, with variations spanning more than two orders of magnitude. As shown in Fig. 9d, it is evident that the thermal conductivity of CNTs bundles arranged in an aligned manner is significantly higher than that of other bundle structures [171].

Additionally, enhancing the interfacial interactions between tubes to increase thermal conductivity is also an effective approach. For instance, methods such as sintering and covalent bonding have been found to increase thermal conductivity with the rise in inter-tubular covalent bonds. However, excessive modification can lead to a reduction of van der Waals forces between the tubes and an increase in phonon scattering [181,182]. Interestingly, as shown in Fig. 9e, Qiu *et al*. [172] proposed a theory of low-frequency phonon resonance between CNTs. By introducing a secondary medium at the interfacial level (such as polymer molecules, halogen doping, nanoparticles, *etc.*), they affected the low-frequency vibrational state density of CNTs, indicating the introduction of a secondary medium at the interface [183,184].

Therefore, improving the alignment, density, and introducing a secondary medium to reduce the intertube thermal resistance of CNTFs are effective methods for preparing CNTFs with high thermal conductivity. Based on this, we have elaborated the current strategies for thermal conductivity enhancement in Supplementary note 9, which also confirmed that the thermal conductivity of CNTFs prepared by the wet-spinning method is significantly higher than that of the other two preparation methods.

## CONCLUSION AND OUTLOOK

This paper reviews in relative detail the research progress of wet-spinning CNTFs over the past two decades. We depict CNTs conformation evolution during the different stages of spinning with an emphasis on the significance of wet-spinning process regulation for obtaining high-performance CNTFs. Subsequently, we systematically elaborate the underlying thermodynamic and kinetic rationales in sequence of dispersion, phase separation, drafting and heat treatment from three courses of wet-spinning including spinning dope, coagulation bath and post-treatment, providing the linkage of processing, structure and property of CNTFs. Although CNTFs embrace greater superiority than traditional yarns both mechanically and conductively, the exceptional nature of individual CNT has not yet been fully translated to their macro-assemblies, CNTFs. Since the investigation of fundamental mechanisms may provide opportunities for the further optimization of the CNTFs property, we summarize the theoretical basics of CNTFs performance behavior and current enhancement strategies.

**Figure 10.**
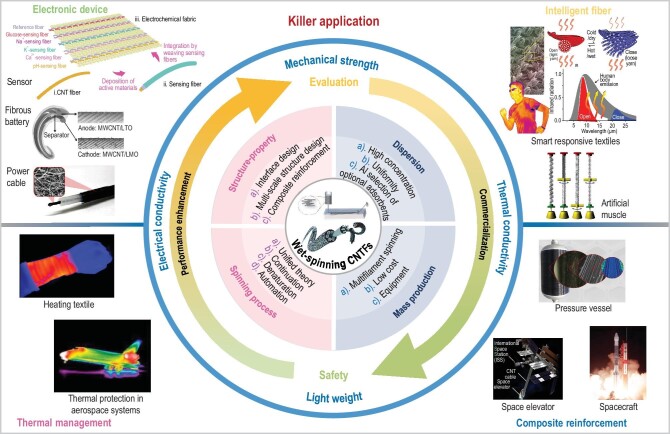
Summary of the challenges faced in the scale-up preparation of CNTFs by wet-spinning and potential future applications. Adapted with permissions from Refs [185–194].

As shown in Fig. 10, CNTFs exhibit excellent mechanical strength, electrical properties, thermal properties and lightness. The multifunctional combined performance of CNTFs grants them a wide range of application scenarios, which include composite reinforcement (pressure vessels, space elevators, spacecraft), electronic devices (sensors, fibrous batteries, power cables), intelligent fibers (smart responsive textiles, artificial muscles) and thermal management (heating textiles, thermal protection in aerospace systems). The transformation of CNTFs from laboratory-scale achievements to practically applicable large-scale products through wet-spinning technology undoubtedly presents challenges. This requires consideration from two aspects. First, the economic feasibility and experimental safety must be assessed from a commercial perspective. There are still several issues waiting to be addressed in order to achieve final large-scale production of CNTFs, such as dispersion (high concentration, uniformity, AI selection of optional adsorbents), massive wet-spinning technique (multifilament spinning) and post-treatment (multi-drafting, thermal-treatment). Second, the structure-property relationship (interface design, multi-scale structure design, composite reinforcement) combined with the spinning process (unified theory, continuation, denaturation, automation) are critical points for fiber performance enhancement. The following will discuss in detail some of the significant issues concerning the future development of CNTFs.

### Appropriate strategy to disperse CNTs

Homogeneous dispersion of CNTs at high concentration is a prerequisite for the preparation of high-performance fibers, and thus the CNTs dispersion mechanism should be further investigated in depth. In terms of the charge transfer mechanism, especially for the superacid system, the corrosivity and volatility of superacids make it difficult to determine the physicochemical properties of spinning dopes, leading to a lack of in-depth understanding of CNTs dispersion states. Therefore more advanced characterization techniques with a customized corrosion-resistant sample chamber and theoretical models are needed to elucidate the formation of supermolecular proton-CNTs complex. In the context of non-covalent adsorption mechanisms, the pivotal factor lies in identifying an adsorbent that exhibits the highest affinity towards CNTs. With evolving advanced algorithms such as artificial intelligence (AI), machine learning, deep learning, *etc.*, it becomes possible to identify the optimal adsorbent among enormous amphiphilic compounds to actualize efficient and highly concentrated dispersion of CNTs with large aspect ratios in aqueous media. The aqueous wet-spinning dispersion confers better compatibility of CNTs with other functional guest components and superior properties can be derived from longer CNTs. Thereby it can further propel the all-round performance enhancement of CNTFs-based applications, such as fiber batteries, fiber sensors and thermal management fabrics.

### Deep understanding of spinning process for CNTFs

Despite the current theories addressing the various stages of spinning, there is still a lack of a comprehensive and systematic rationale applicable to the structural evolution of CNTs from powder to dispersion and finally fiber. Since the wet-spinning process of CNTFs is multivariably-determined and these variables demonstrate an intertwined nexus, making it difficult to summarize the effect of a specific variable on fiber properties. Given the implausibility of effectively controlling the different variables from an experimental point of view, machine learning capable of decoupling various parameters and quantitatively weighing the individual parameters can serve as the resolution. With the ability of processing large amounts of data, it may render fundamental mathematical formula with each parameter having its own impact factor, guiding the fabrication of better performed CNTFs. In the study of CNTFs, computational methods and simulation calculations are widely utilized to give deep understanding for CNTFs performance enhancement. For instance, numerous models were established to investigate the mechanical properties of CNTFs [136,155]. The correlation between load transfer in CNT networks and cross-link density was also explored by the graph theory [195]. In addition, machine learning techniques have also been integrated to accelerate the complex and time-consuming molecular simulation, which showcases the capability of predicting the quantum-mechanical energies of CNTs [196]. For instance, machine learning has been employed to investigate the interfacial shear strength at a molecular scale [197], which has led to significant improvements in the performance of CNTs-reinforced composites. Beyond the fusion of machine learning with simulation calculations, there is an emerging trend of building automated laboratories equipped with AI to drive self-updated experimental routes [198]. Apart from the investigation of spinning rationale, polymer compositing is an important avenue for CNTFs advancement. It is still obscure as to why composite showcasing has even better properties than pure CNTFs. Whether it is the polymer that affects the rheological properties of the spinning dopes and thus promotes fiber orientation, or the CNTs inducing crystallization of the polymer segments, it is not clear which endows the fiber with high mechanical performance. Or alternatively, maybe it is the polymer filling the voids that results in enhancement of intertube interactions or maybe there are several effects that exist simultaneously to synergistically strengthen the fiber properties. The unmet need is to systematically study and categorize these enhancement mechanisms and determine the extent to which each mechanism contributes to the enhancement of the composite properties.

### Comprehensive resolution of the structure-property relationship

Resolving the structure-property relationship between CNTFs’ microstructure and mechanical-electrical-thermal properties is a fundamental and urgently needed task. The interface structure should be dissected primarily since it is the restrictive factor and many studies have shown that CNTFs properties are highly contingent on interfacial interactions. A compatible interfacial interaction not only promotes the intertube load transfer and thus enhances the mechanical properties, but also facilitates the rate-determining step-interfacial thermal transport (ITT) and interfacial electrical transport (IET) during conduction, thus effectively boosting the carrier transport. Although the basic understanding for structure-performance relationship of CNTFs and their critical parameters has been summarized. However, from atomic-level CNTs to microscopic CNT bundles to macroscopic CNTFs the connection has not been fully established, which needs further examination in the future.

### Establishing standard characterization and evaluation systems

To allow amenable assessments of CNTFs properties and designed wet-spinning process, the field must standardize the characterization methods and articles should provide some indispensable parameters since the performance of CNTFs is closely reliant on the raw CNTs properties. We summarized the basic characterization techniques as well as the performance evaluation strategies in Table S1.

From the aspect of raw CNTs, (1) aspect ratio is the overarching parameter since the fiber strength is proportional to the aspect ratio of CNTs. Thus, this parameter should be provided and methods including atomic force microscopy (AFM), transmission electron microscope (TEM) and viscosity can be adopted. (2) Defect is also an influential ingredient for the well-dispersion of CNTs and load transfer within fibers. It also serves as a scattering point of carrier and phonon transport, reducing electrical and thermal conductivity simultaneously. Raman spectroscopy and TEM is a feasible instrument to obtain the information. (3) Impurity mainly refers to the amorphous carbon and metal catalyst. TGA allows direct measurement of impurity mass fraction which should also be incorporated in the article. Meanwhile, energy-dispersive X-ray spectroscopy (EDS) can also present the distribution of metallic elements. From the aspect of spinning dopes, the dispersion state and LC phase are critical for the comparison and assessment of different CNTFs. POM image and shear viscosity are fundamental statistics to describe CNTs dispersion state and phase condition while neutron scattering, X-ray scattering and cryo-EM can further underpin the conclusion. For CNTFs, tensile strength (maximum force over area, GPa) is frequently used in publications to describe mechanical properties. However, due to the voids in the fiber cross-section and inhomogeneities in the axial direction of the fibers, it is challenging to determine a representative cross-sectional area. Therefore, specific strength (maximum force over linear density, N tex^–1^) is preferred in the textile industry since it removes the density effect and the linear density is easier to determine accurately than the cross-sectional area, yet a GPa unit can combine most of the literature and make meaningful assessment. Here, we suggest that researchers should provide more detailed statistics for standard evaluation and readers can calculate for themselves to get the numbers they want, e.g. maximum force (N), linear density (tex), density (g cm^−3^) and cross-section area (m^2^). Kelvin four-terminal sensing is recommended for the electrical conductivity characterization of fibrous material while 3ω method, steady-state dc thermal bridge method and T-type method are viable in thermal aspects.

### Mass production from CNTs ingredient to spinning production line

The prerequisite for commercializing CNTFs and facilitating their societal impact hinges on the capability for large-scale production. Among the prevalent techniques, the wet-spinning technique inherently demonstrates superiority in mass production due to its compatibility with conventional industrial fiber manufacturing. Currently, the global annual production capacity for high-quality CNTs is approximately a thousand tonnes per year. Accompanying the increase in yield, there has been a notable reduction in the cost of MWCNTs raw materials, from ․150 000 per kilogram to less than ․50 per kilogram, while SWCNTs have similarly seen a price reduction to ∼․1500 per kilogram at the tonne scale. It is anticipated that the price of SWCNTs will further decrease with an escalation in production volume in the near future. To genuinely realize the large-scale production of CNTFs, it is critical to overcome the challenges associated with the thousand-level multifilament fiber production techniques. There exists a pressing need for comprehensive systematic studies that furnish theoretical guidance for the multifilament preparation process of CNTFs. In practical terms, scaling the number of filaments introduces significant complexity into the preparation system. Variations in the hydrodynamic distribution at the point of extrusion, specifically the spinnerets, manifest substantial differences. Furthermore, the flow dynamics of the spinning solutions and the differential shear stresses experienced by individual spinnerets can lead to inconsistent extrusion rates, resulting in potential clogging and breakage of the nascent filaments. These issues present significant obstacles to achieving a continuous, reliable process for the preparation of multifilament CNTFs. Furthermore, the complexity associated with the dual diffusion in multifilament systems is intensified due to the significant discrepancy in the coagulation bath composition between the peripheral and central zones of a multifilament bundle, underscoring the need for advanced theoretical models to guide the coagulation process of multifilamentary CNTFs. Additionally, while CSA stands out as the leading dispersant for fabricating high-performance CNTFs, the hazardous nature and elevated cost of CSA, alongside its coagulant counterpart, acetone, pose substantial barriers to the industrial scale-up of high-performance CNTFs. These challenges necessitate the exploration for alternatives, effectively translating into the requirement for the identification of an optimal adsorbent. This identification process could significantly benefit from the utilization of sophisticated algorithms powered by AI, which could streamline the search for viable dispersant solutions.

### Proper solution for safety issue during large-scale production

The toxicity and environmental persistence of CNTs has been in the limelight of public concern along with increasing CNTs utilization [199–201]. To date, there has not been a putative conclusion as to whether CNTs will impose threats to human health. Unfortunately, the World Health Organization's International Agency for Research on Cancer (IARC) incorporated MWCNTs as a Group 2B carcinogen in 2014. Afterwards, Chemsec called for the replacement of CNTs and lists them at ‘substitute it now’ in 2020 [202]. The most substantial way in which CNTs cause impairment to the human body is through respiration. An estimation of 0.2-2 μg m^–3^ CNTs inhalation will bring about excess risk of lung disease. Nevertheless, the toxicology research is mainly fixated on MWCNTs seeing their resemblance to asbestos fibers in physical dimensions. While for SWCNTs, which is commonly used for CNTFs fabrication, the existing evidence cannot suffice for the denouncement of their carcinogenic effects [203]. It was found that kidneys are capable of purging SWCNTs rapidly regardless of their giant size for renal filtration. Their inhalation can also be obviated by correctly wearing protective equipment. Moreover, in the form of 1D macroscopic assembly, CNTFs, CNTs are intensively cohered and ascribe to a strong van deer Waals interaction thus showing little propensity of falling out. This integral unity is regarded as chemically stable and thus can be used in various circumstances. Also, using bio-compatible and inertial polyurethane or polydimethylsiloxane to encapsulate CNTFs-based devices can alienate the intimate contact of both and further inhibit the potential personal hazards of CNTs. To recapitulate, fabrics or electronics based on CNTFs can be perceived as living organism–harmless, but this requires more toxicological studies on humans or higher mammals.

### Killer application for CNTFs

Encompassing the excellent properties in various facets, such as light weight, high flexibility, mechanical strength, electrical and high thermal conductivity, CNTFs ought to suffice the demands in advanced fields. The last decade has witnessed stunning achievements of this high-performance fiber in various fields. Using its lightweight but high strength mechanical properties, CNTFs can serve as reinforcing materials applied in aerospace, defense, industry and other cutting-edge fields. Artificial muscles, fibrous electronics, flexible sensors, energy storage, *etc.* can also be realized by CNTFs characterized by their excellent mechanical and electrical properties. In addition, in the field of thermology, it can be used as thermal protection materials in extreme environments such as spacecraft radiators and as a foundation for smart thermal management profiting from the high thermal conductivity. However, the mechanical strength of T1200 CFs produced by Toray scale at 8 GPa, the thermal conductivity of graphene fiber can reach 1580 W m^–1^ K^–1^ through systematic thermal treatment [7]. Therefore, to realize the market-oriented development in the future, on the one hand CNTFs necessitate further study in a bid to advancing their overall properties that can compete with or even surpass those high-performance fibers. On the other hand, there is still a lack of specific application scenarios that can integrate the various outstanding properties of CNTFs. It is of great importance to find a killer application belonging exclusively for CNTFs that can capitalize on their excellent properties in various facets e.g. light weight, high flexibility, mechanical strength, electrical and high thermal conductivity.

## Supplementary Material

nwae203_Supplemental_File
