# Supplementary material for: Wet-spinning of carbon nanotube fibers: dispersion, processing and properties
Source: Natl Sci Rev. 2024 Jun 12;11(10):nwae203. doi: 10.1093/nsr/nwae203 (PMC11409889; doi:10.1093/nsr/nwae203)
Supplement: nwae203_Supplemental_File [file nwae203_supplemental_file.pdf]

Supporting Information

Wet-spinning of Carbon Nanotube Fibers: Dispersion, Processing and Properties

Zhicheng Yang[1,2,6,7], Yinan Yang[2,7], Yufei Huang[3], Yanyan Shao[4], He Hao[3], Shendong Yao[5], Qiqing Xi[1], Yinben Guo[1], Lianming Tong[3], Muqiang Jian[6], Yuanlong Shao[2,5,6,*], Jin Zhang[2,3,5,6]

[1]School of Materials Science and Engineering, Shanghai University of Engineering Science, Shanghai 201620, China;

[2]School of Materials Science Engineering, Peking University, Beijing 100871, China;

[3]Center for Nanochemistry, Beijing Science and Engineering Center for Nanocarbons, Beijing National Laboratory for Molecular Sciences, College of Chemistry and Molecular Engineering, Peking University, Beijing 100871, China;

[4]College of Energy Soochow Institute for Energy and Materials Innovations (SIEMIS), Key Laboratory of Advanced Carbon Materials and Wearable Energy Technologies of Jiangsu Province, SUDA-BGI Collaborative Innovation Center, Soochow University, Suzhou 215006, China;

[5]Academy for Advanced Interdisciplinary Studies, Peking University, Beijing 100080, China;

[6]Beijing Graphene Institute (BGI), Beijing 100095, China

[7]These authors contributed equally.

***Corresponding author**. E-mail: shaoyuanlong@pku.edu.cn

Supplementary note 1. Truncation effect of sonication and corresponding dispersion enhancement strategy

Lu *et al.*[1] have studied the effect of ultrasonication time on the dispersion state of carbon nanotubes (CNTs) including bundle size, tube length, and their mechanical performance of spun fibers. CNTs spinning dope of 0.6 wt% with sodium deoxycholate (SDOC) used as surfactant was injected into a mixed coagulant of isopropanol and deionized water (3:1). As summarized in Figure S1a, the viscosity of dispersion increased significantly after ten minutes sonication, because the ultrasonication input energy caused the originally agglomerated CNTs to be uniformly dispersed. Meanwhile, the strong interaction between CNTs also increased the dispersion viscosity. Accompanied by the further extension of sonication time, the dispersion viscosity gradually decreased, which was attributed to the shearing slippage of CNTs and length shortening. In addition, the size of nanotube bundle also tends to decrease with the increase of sonication time as directly observed by high resolution transmission electron microscope (HR-TEM). After a synergetic balancing of drawbacks and superiorities induced by sonication, thirty minutes treatment yields the fiber with the most robust mechanical properties. It embraces modest entanglements and voids derived from thin bundles alongside effective load transfer derived from relatively intact tube length.

Therefore, the pivot on this research domain is how to realize a high concentration of CNTs dispersion with preserved tube length. Since the CNTs length is majorly spoiled by sonication, further discussion regarding ultrasonic treatment minimization or replacement will be provided as follow. Among all influencing parameters, the surfactant type is the primary influencing role should be earnestly considered. A comprehensive quantitative assessment of dispersion ability including oligonucleotides, peptides, lignin, chitosan, and cellulose and surfactants such as cholates, ionic liquids, and organosulfates in aqueous solution have been also provided by Gilman[2]. It offered a guideline for the selection of the most effective dispersent. With focusing on

cholate type surfactant, Yumura *et al.*[3] corroborated the best surfactant-tube affinity of sodium taurodeoxycholate (TDOC), which contributed to the finest fiber morphology and shortest period of sonication time indicated from Figure S1b-d. In such a scenario, 30 min sonication of TDOC-CNTs dispersion yielded finest fiber (Figure S1b). However, elongating sonication time to 40 min is favorable by dispersants with low affinity to CNTs like SDOC to give the comparatively compact fiber (Figure S1d). In contrast, prolonging sonication time only rendered the fiber obtained from TDOC-CNTs dispersion to a loose structure and insufficient internal binding force as shown in Figure S1c. Inferred from Figure S1e, in tandem with the fiber morphology, the carbon nanotube fibers (CNTFs) prepared from 30 min sonication of TDOC-CNTs dispersion prevail over all counterparts in Young's modulus and the other typical properties. To our knowledge, the strongest CNTFs to date produced from surfactants assisted wet-spinning dope was TDOC-CNTs dispersion derived fiber with optimized surfactant-tube affinity and sonication time. Pre-treatment is also an essential procedure, which was designed to diminish the ultrasonication time. Incorporating oleum (20% excessive SO_3) pretreatment and novel condensation methods, *i.e.* dialysis, CNTs dope with 1.8 wt% was obtained, which is the highest concentration ever obtained *via* aqueous solution spinning technique. Based on the earlier work about superacid spun single-walled CNTs (SWCNTs) fibers, residual acid could lead to intercalation within the SWCNTs, which even lead to the partial exfoliation of CNTs bundles[4]. The subsequently fabricated CNTFs exhibited a tensile strength approaching to 1 GPa and Young's modulus of 123 GPa, which exceeds those made from conventional surfactant stabilized CNTs dispersions[5]. Moreover, researchers also devoted great efforts to avoid the CNTs shearing induced by sonication by trial of different means of dispersion. Welland *et al.*[6] have employed a microfluidization dispersion process to facilitate the high-concentration (up to 20 mg mL^{-1}) CNTs dispersion using carboxymethyl cellulose sodium salts as a non-covalent absorbent. After the thermal decomposition of carboxymethyl cellulose sodium salts, the CNTFs were replenished with Cu by periodic pulsed reversed electroplating process. Based on the retention of multiwalled CNTs (MWCNTs) length *via* replacing

sonication, even after the intermix of 60 % Cu (the strength is generally below 300 MPa), the resultant MWCNTs fibers could exhibit a tensile strength of 318 MPa with a modulus of 53.78 GPa. Meanwhile, a fascinating specific electrical conductivity of 9.38×10^4 S cm^2 g^{-1} is achieved, which is even superior to Cu wires (6.6×10^4 S cm^2 g^{-1}). Wang *et al.*[7] also substitutes the sonication step with superacid-surfactant exchange to treat intact CNTs, in which the direct neutralization of SWCNTs-superacid solution in the presence of a surfactant gives the uniform dispersion formation. The resulting macroscopic assembly consisted of 350 % longer CNTs than the counterpart of the sonicated one, which manifests a better electrical conductivity.

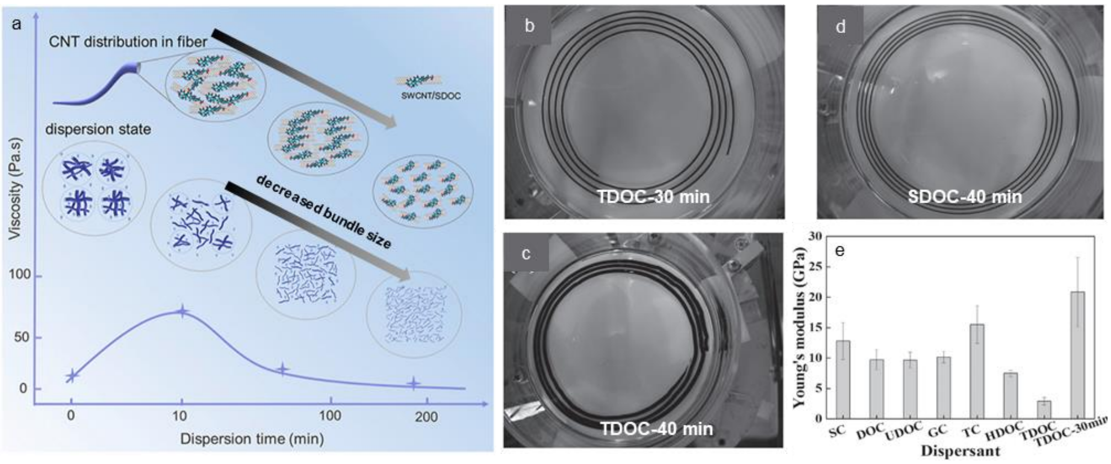

Figure S1. (a) Effect of ultrasonication time on the viscosity of spinning dopes indicating the dispersion state of CNTs. Bundle size is decreased and tube length is truncated by the ultrasonication treatment. Adapted with permission from ref.[1]. Copyright 2021 Elsevier. (b-c) Digital photographs of fiber spun from (b) TDOC with 30 min ultrasonication treatment, (c) TDOC with 40 min ultrasonication treatment, and d) SDOC with 40 min ultrasonication treatment. (e) Effect of different sonication times and surfactants on the spun fibers Young's modulus. Adapted with permission from ref.[3]. Copyright 2017 IOP Publishing on behalf of the Japan Society of Applied Physics (JSAP).

In addition, ethylene glycol (EG)[8], ionic liquids[9] and many natural amphiphiles such as DNA[10,11], hyaluronic acid[12], chitosan[2] and protein such as lysozyme[13,14] have been also utilized for CNTs dispersion. These

bio-macromolecules stabilizes CNTs by wrapping around the individual CNT with amphipathic molecules, which simultaneously increasing inter-tube repulsion.

Supplementary note 2. Characterization of Protonation process

Specifically, the UV-vis-NIR absorption spectra of CNTs dilute dispersion in various superacids provided by Smalley *et al*.[15], are featured by a broad absorption ranging from 700 to 1100 nm. This absorption is more analogous to a charge-transfer process of SWCNTs solubilized in carbon disulfide[16] or when doped with Br_2[17] at 1.07 eV (1158 nm). Moreover, v_1 to c_1 transitions of SWCNTs are sensitive to the CNTs surface adsorption. This transition is reflected as the first van Hove transition. Given the majority of v_1 valence electrons are depleted during the charge-transfer process, the first van Hove transition is smeared in the UV-vis-NIR spectra. As shown in Figure S2a, the Raman spectra with different excitation wavelengths also confirmed this explanation[15]. Raman signal obtained from 780 nm laser stimulation is in resonance with the first van Hove transition involving v_1 valence electrons. There is no distinct signal observed from the consequential Raman spectra after dispersed CNTs in chlorosulfonic acid (CSA), as v_1 valence electrons are removed by the strong acid. It presented a significant difference compared to pristine dry sample under the same laser source (line b in Figure S2c). In comparison, deeper lying v_2 valence electrons excited by 514 nm laser source, which exhibits a distinguishing absorption peak (line c) at 1619 cm^{-1} for SWCNTs-CSA hybrid paste. A blueshift of 23 cm^{-1} *cf.* line a indicates a positive charge be shared by approximately 14 carbon atoms calculated from Eklund's model. Based on the above spectroscopic results, the v_1 valence electrons on the CNTs are stripped, resulting in their partial polarization with a certain positive charge.

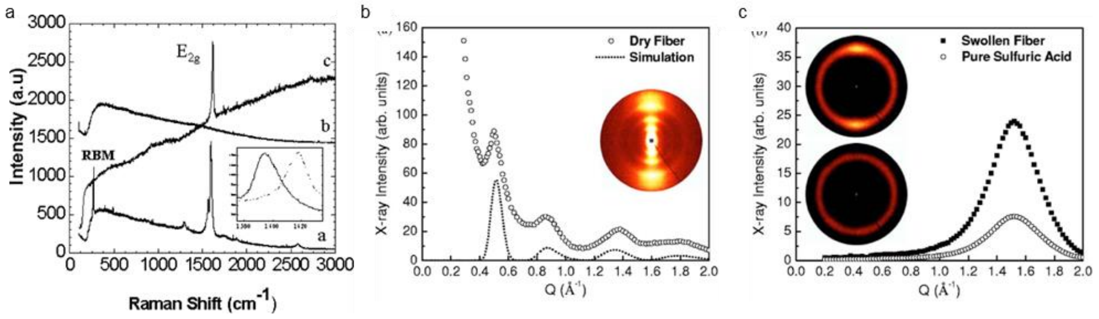

Figure S2. (a) Raman spectrum of the dry SWCNTs sample excited by 780 nm labeled as line a, SWNT-chlorosulfonic acid paste (2.5 wt/wt%) recorded with the same

excitation source labeled as line b, Raman spectrum of the SWNT-chlorosulfonic acid solution (2.5 wt/wt %) recorded with the argon ion laser (λ_{exc}) 514 nm as exciting source, labeled as line c. Adapted with permission from ref.[15]. Copyright 2004 American Chemical Society. (b) and (c). X-ray scattering data for dry fiber and its simulation result (b) and swollen fiber and pure sulfuric acid (c). Adapted with permission from ref.[18]. Copyright 2005 American Physical Society.

To further dissect protonation mechanism, the specific physical profile of the CNTs interacting with the super acid has been further characterized[18]. With the various sulfuric acid concentrations, the SWCNTs fibers tend to swell and sink when immersed in 102% or 120% sulfuric acid. In contrast, CNTFs exposed to 96% sulfuric acid floated at the surface, which indicates that the 96% acidity sulfuric acid is inadequate because proton cannot penetrate into CNTFs. However, the simply infiltration of sulfuric acid into accessible voids inside fibers is unreasonable to paraphrase the sinking behavior, which further given the density discrepancies between fibers (<1.1 g cm^{-3}) and sulfuric acid (*ca.* 1.9 g cm^{-3}). The swollen fibers' density should outweigh sulfuric acid. The higher long-range ordered sulfuric acid solids (2.13 g cm^{-3}) density implies that there is bound to be an interaction between the CNTs and sulfuric acid. A charge-transfer complex is formed between the CNTs and sulfuric acid molecules, which triggers a locally ordered sulfuric acid molecular layer near CNTs. Figure S2b and c demonstrate the comparison of X-ray scattering data between dry SWCNTs fibers and fibers swollen by 102% sulfuric acid[18]. In support of this protocol that distinct Bragg reflections pertinent to aligned CNTs and the scattering intensity at small angles originated from charge distribution heterogeneity between fibers and voids both are smeared. Acid permeation in a disorder manner accounts for the disappearance of Bragg reflections since the triangular lattice of CNTs is disrupted. While the absence of small-angle X-ray scattering (SAXS) intensity showing the homogeneity of the swollen fibers, namely, conformability between CNTs and acids. However, by virtue of the electron scattering length density (SLD) variation between fibers (~1.29) and sulfuric acids (1.64), necessarily there ought to be an interaction between the two that alters their charge distributions to reach homogeneity. Furthermore, an anisotropic scattering

pattern in Figure S2c stands out referring to pure acids with about the same orientation degree (FWHM = 32.1°) to that of CNTFs. Implying the origin of the anisotropic scattering is derived from cylindrical annulus of sulfuric acids around CNTs.

The partially organized sulfuric acid layers were also verified by the differential scanning calorimetry (DSC)[19] that the phase transition of sulfuric acid is drastically altered with regards to SWCNTs contents. It is assumed that free sulfuric acid molecules are bonded after the introduction of CNTs and the bounded part of sulfuric acid will not participate and offer no contribution to the phase transition enthalpies. Whereupon, increasing SWCNTs concentration brought about deceasing trend in melting point and enthalpy. In contrast, SWCNTs displays no perceivable effect on the phase transitions of benzene or water showing marginal interactions between them.

This far, a coarse protonation profile is rendered, the negatively-charged electron-rich backbone of CNTs allures the protons ionized from superacid establishing strong interactions *via* electron-cloud polarization. This subsequently causes the partially positively charged CNTs to attract the counterions through electrostatic attraction, ultimately leading to an ordered acid layer formation around the CNTs. However, its precise physical depiction still merits further studies to characterize it, including the specific surrounding anion distribution, the number of ordered acid layers, and the contribution of the anion in providing steric hindrance effect to thermodynamically stabilize the dispersion.

Supplementary note 3. Characterization of CNTs phase behavior in superacid

The CNTs phase behavior in superacid was initially studied *via* rheologic and optical characterizations in 2004[20]. In detail, with the accumulation of CNTs concentration, the dispersion state gradually morphed from individual CNT Brownian[21] rod-like phase to a distinctive 'spaghetti-like' nematic state. This nematic phase consists of axially mobile tubes while perpendicularly fixed strands in some regions, which is in equilibrium with a dilute isotropic phase. Finally, the dispersion forms into a full-blown polydomain nematic liquid crystal(LC). Shortly thereafter, a convergence of centrifugation treatment and UV-vis-NIR spectroscopy have been developed to quantitively evaluate the isotropic-nematic phase transition threshold (φ_c) of SWCNTs in varied acidity superacids[22]. According to Flory's prediction[23], the appliance of external force fields precipitates biphasic dispersions of rigid-rod polymers into isotropic and ordered phases. This theory maybe also applicable for CNTs in superacids. Biphasic SWCNTs-superacid dispersions were treated with centrifugation to achieve isotropic phase and dense nematic phase separation. SWCNTs concentration was quantified *via* UV-vis-NIR absorption spectroscopy, based on linearly light absorbance with concentration variation. It can precisely match up with Beer's law in the range from 400 to 1400 nm for SWCNTs-superacid dispersion supernatant after stoichiometrical dilution at low SWCNTs concentration (below 200 ppm). With quantitative determination of φ_c, a concise phase diagram was postulated to provide laconic information. The chimney of biphase dilates with decreasing acid strength, which is mainly attributed to the demeaning of critical isotropic-nematic phase transition concentration. However, CNTs interactions in superacids were still mysterious.

The phase behavior of CNTs wet-spinning dope is also characterized *via* a series of advanced techniques. Based on small-angle neutron scattering (SANS), long CNTs in nematic phase presents a 2D lattice expansion in dilute state, whereas short CNTs in

biphase seem to exhibit an intermediate expansion between 2D and 3D. In addition, identification of LC phase with higher order, columnar phase as depicted in Figure S3a, was realized by virtue of SAXS. Indicated from the Figure S3b, peak position is proportional to the packing density and positional ordering that CNTs dispersion with higher volume fraction bears more compact and aligned structure. Moreover, relative position of the second peak with respect to the first peak for the scattering signals at higher concentration is the key parameter to characterize the hexagonal packing of columnar phase[24].

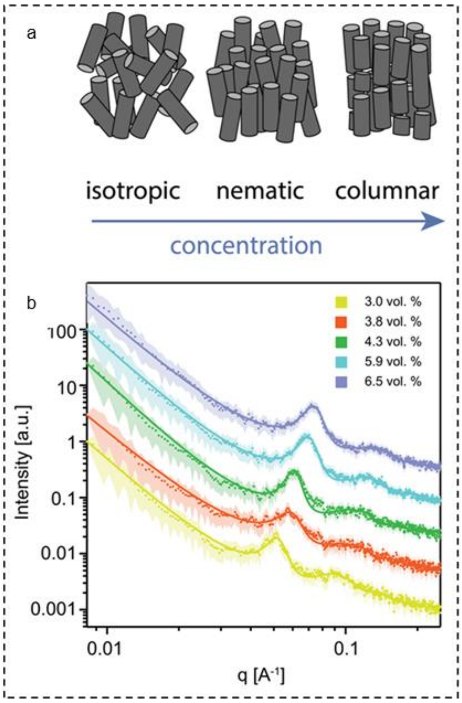

Figure S3. (a) Phase evolution from isotropic to nematic and finally columnar along with the increasing of CNTs concentration. (b) SAXS measurements for CNTs-CSA solutions at concentrations ranging from 3 to 6.5 vol% fitted to a hexagonally close-packed cylinder model. Adapted with permission from ref.[24]. Copyright 2021 Royal Society of Chemistry.

Supplementary note 4. The thermodynamics of the phase separation process

As for the thermodynamics of phase separation process shown in Figure S4, in a typical ternary phase diagram of polymer (P), solvent (S), and coagulant (N), there exists a two-phase region versus a homogeneous region[25,26]. During the solidification process, the filament composition gradually changes along with the continuous solvent and coagulant dual diffusion. Starting from the initial spinning dispersion (SD), the composition change route and the angle θ between S-P depends on the mass transfer flux ratio of solvent and coagulant (J_s/J_N, J_s and J_N represent the mass transfer flux of solvent and coagulant, respectively). The analysis can be divided into four regions. Region 1 ($-\infty < J_s/J_N \leq u^*$), the dispersion gradually dilutes without undergoing phase transition, which leave the dispersion in a homogeneous state. Region 2 ($u^* < J_s/J_N \leq 1$) is the region that CNTs filament gel starts to phase separate and solidify from the dispersion. However, due to the coagulant diffusion rate is still slower than solvent, the derived CNTFs still demonstrate uneven surface morphology with relative low density. Region 3 ($1 < J_s/J_N \leq u^{**}$), as the solvent diffusion rate accelerates due to the concentration gradients, the phase separation and filament solidification move to a deeper level, which result in more uniform and compact fiber configuration. Region 4 ($u^{**} < J_s/J_N \leq \infty$), the fiber could form into uniform and densified configuration. However, such dual diffusion condition is demanding for coagulant and solvent selection. In general, the wet-spinning process is usually located in Stage 3. The closer the solidification route is to Stage 4, the better the microstructure and properties of the fiber.

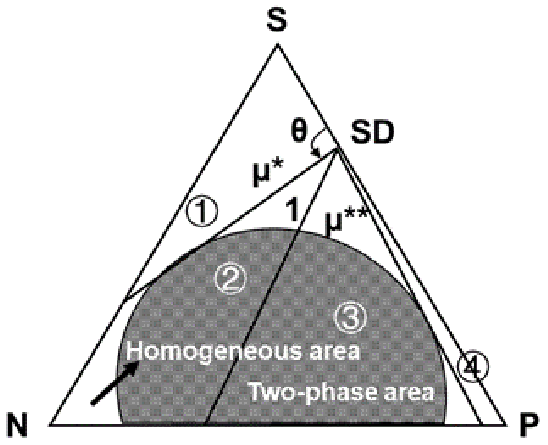

Figure S4. Ternary phase diagram in solution spinning process.

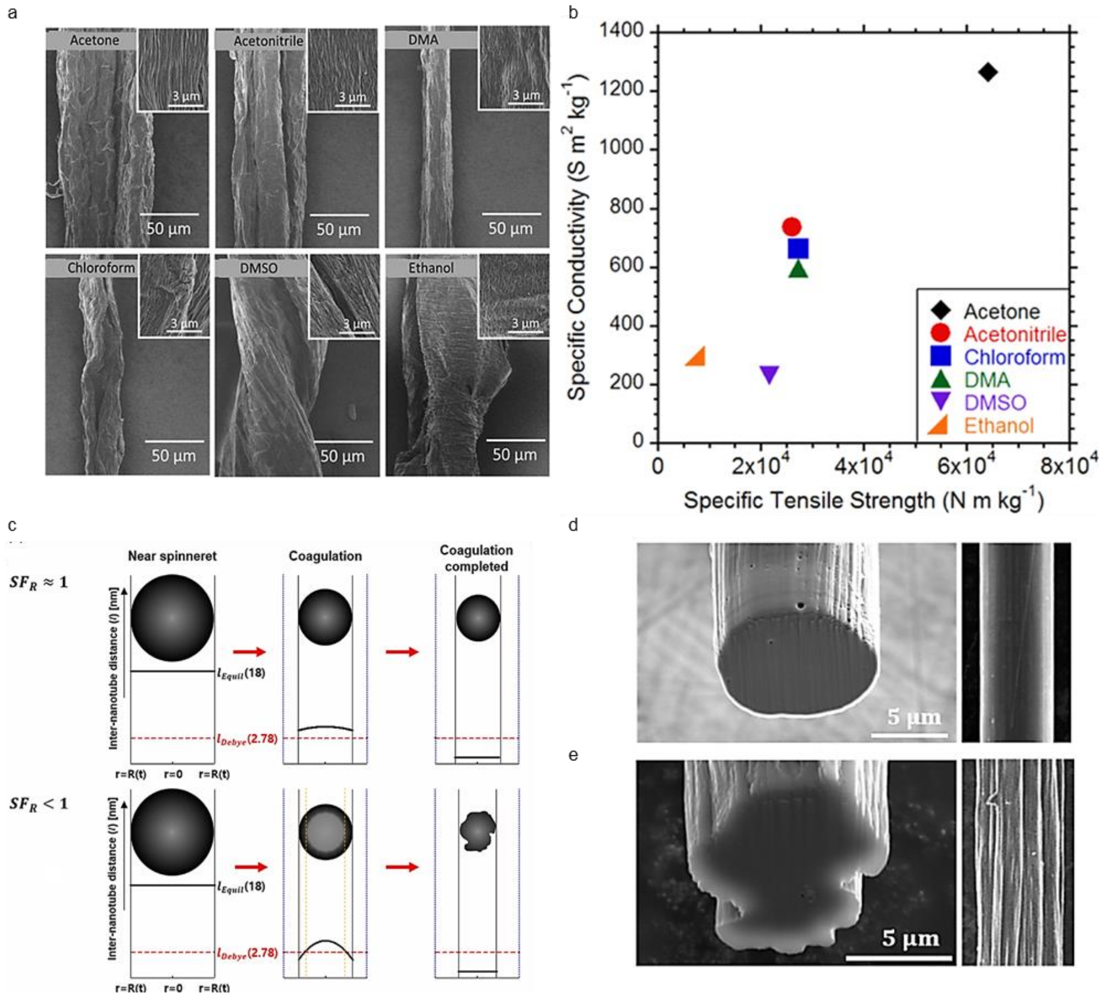

Figure S5. (a) Morphology and structure of fibers solidified in different coagulant. (b) Properties of SWCNTs fibres extruded into various composition coagulant baths analyzed by specific conductivity and specific tensile strength. Adapted with permission from ref.[27]. Copyright 2015 American Chemical Society. (c) Changes of inter-nanotube distance (l) during the coagulation with SF_R~1 and SF_R□1. (d) SEM structure of perfect circular fiber with SF_R~ 1. (e) SEM morphology and structure of imperfect circular fiber with SF_R□1. Adapted with permission from ref.[28]. Copyright 2022 Elsevier.

Supplementary note 5. Detail drafting process for enhancing CNTFs

Figure S6a and b summarize the relationship between the drafting parameters D_R, D_R^*, and derived fiber structure features (fiber orientation factors (S), voids/defect density, fiber packing density *etc.*). Thus, the drafting process not only reshapes the geometry of the nascent fibers but also optimizes the derived fiber microstructure, chain orientation and crystallization. Lee *et al.*[29] explored in detail that the orientation is an important factor in the preparation of highly conductive CNTFs. They propose a Hyper-Spin-Line (HSL) model for obtaining well-aligned CNTFs, which predicts the minimum stretch ratio (D_R^0) for CNTFs to achieve target orientation at different bottom bola numbers (De) (Figure S6c). They concluded that within a stable operating window, the CNTFs derived from the rational ratio D_R (larger than D_R^0) can pragmatize the target S close to 1 (Figure S6d). Utilizing this model, predicted a minimum draw ratio D_R^0 of 1.66 and specific strengths of up to 100 cN tex^{-1} for D_R greater than 1.66 at different concentration levels of 0.8 wt%, 1.4 wt%, and 2.0 wt%[28,29]. Obviously, drafting is the critical process that determines the orientation degree of CNTFs.

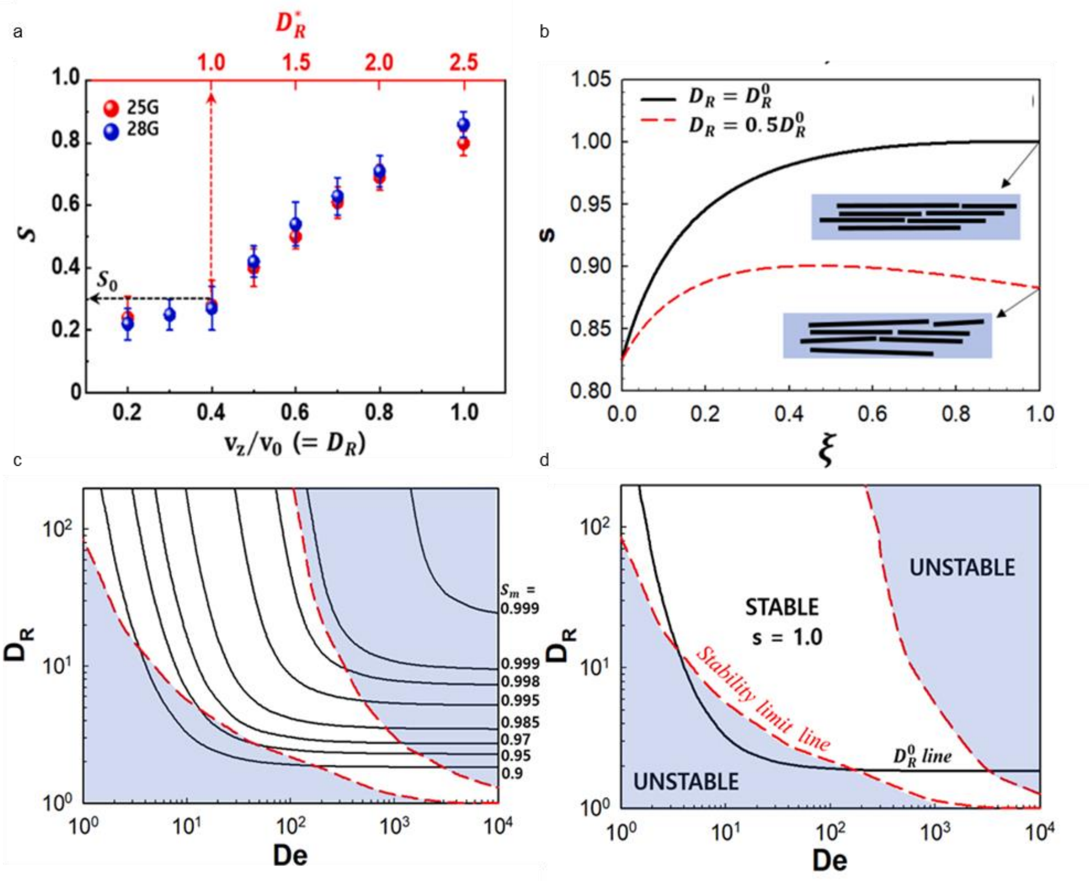

Figure S6. (a) Corrected draw ratio (real draw ratio, D_R^*) obtained from D_R^* change of initial orientation factor (S) with decreasing the winding rate (v_z). Adapted with permission from ref.[28]. Copyright 2022 Elsevier. (b) Effect of draw ratio (D_R) on the evolution of the orientation of CNTs in the spin line; (c, d) Best processing window for preparing fiber with $S = 1.0$ and $C_a = 0.9$; (c) $S_m = 0.9$, (d) for $S_m \geq 0.9$. Adapted with permission from ref.[29]. Copyright 2021 Elsevier.

Subsequently, Ku *et al*.[28] utilized this model to systematically study the draw ratio variation effects on the fiber microstructure, especially the inter- and intra-bundle voids evolution during wet-spinning. Fiber orientation sprawling can hinder the axial interfacial contact between CNTs and voids formation. The volume fractions of the CNTFs internal voids at different draw ratios have been characterized by X-ray microscopy (XRM) (Figure S7a). It can be observed from Figure S7b that the porosity decreases continuously by 97% along with the increase of D_R. Figure S7c indicates that the orientation factors obtained from the experiments match well with the HSL mode simulation results. It is evident that the fiber morphology and structure are significantly

affected by the draw ratio increase. The analysis of the performance results in Figure S7d and e demonstrates an 80% improvement in conductivity and tensile strength after drafting treatment. Therefore, it can be concluded that drafting can gradually optimize the microstructure and macroscopic morphology of CNTFs, by which ultimately achieve high-performance CNTFs with high orientation, low defects and low porosity.

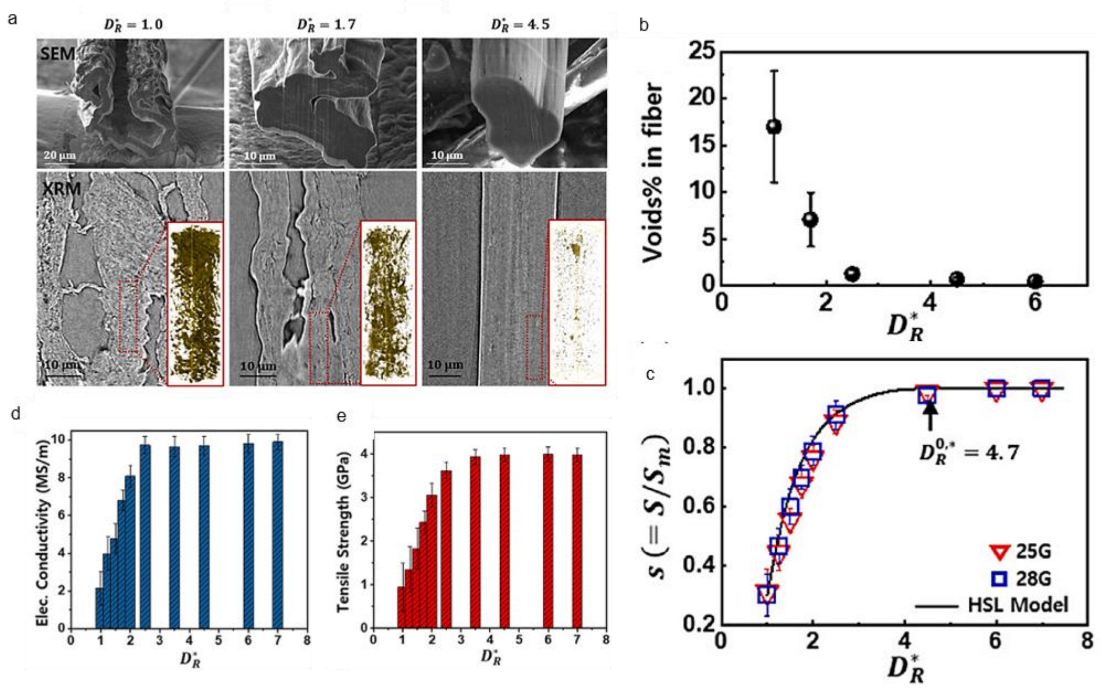

Figure S7. (a) D_R^* = 1.0, D_R^*= 1.7, and D_R^* = 4.5, In XRM images, white is CNTs, and yellow is internal voids; b) Volume% of void defects in fiber depending on D_R^*. (c) The dimensionless orientation factor (S) of CNTFs depending on the real draw ratio (D_R^*). Relationship between the draw ratio and orientation factor for various needle diameters, and comparison with HSL model. (d, e) The dependence of draw ratio on tensile strength and electrical conductivity. Adapted with permission from ref.[28]. Copyright 2022 Elsevier.

Supplementary note 6. Carbon fibers heat treatment process

The heat-setting refers to the process of eliminating the intrinsic stress that exists in the fibers after drafting. Due to the different heat and stress fields to which the fibers are subjected during processing, there are non-uniform cavities. The molecular chain

segments inside the fibers are in a partially relaxed and partially strained state, resulting in the presence of uneven internal stresses in the fiber structure. To eliminate the internal stress, it is necessary to select a suitable heat-setting temperature, which is usually slightly higher than the glass transition temperature. Pre-oxidation is also known as heat stabilization, polyacrylonitrile fibers are generally pre-oxidized in the temperature interval of 180~300℃. The pre-oxidation includes the reactions of cyclization, oxidation, dehydrogenation and thermal decomposition, in which the molecular chain could be crosslinked to form a trapezoidal structure. Such structure is resistant to the high temperature treatment, which can avoid fusion and melting during the fiber carbonization. It could also increase the carbon utilization rate. The pre-oxidation is one of the key steps for the preparation of high-performance carbon fibers (CFs). The carbonization process generally includes thermal decomposition, and thermal condensation with the inert gas protection (nitrogen or argon), in which remove hydrogen, oxygen, nitrogen and other elements results in forming a graphite-like structure with a carbon content of 92% or even more, with transforming from a trapezoidal structure to a chaotic layered graphite structure. Carbonization is generally divided into low-temperature carbonization stage (300~800℃) and high-temperature carbonization stage (900~1600℃), in which the regulation of carbonization temperature, carbonization time and carbonization tension can influence the fiber structure evolution. The graphitization process is usually heat-treated in argon gas at 2200~3000 ℃. The chaotic layered graphite structure of the CFs gradually develops into a three-dimensional ordered graphite structure. The high modulus and high thermal conductivity of CFs are strongly associated with the microcrystalline structure. As the graphitization temperature increases, the graphite microcrystalline size and orientation are gradually enhanced, with reduction of the entanglement and cross-linking between graphite lamellae. Meanwhile, the spacing between graphite layers decreases while increasing the modulus as well as the thermal conductivity of derived fibers[30].

Supplementary note 7. Mechanical enhancement of CNTFs

CNTFs can afford more load through minimizing the fiber cross-sectional area, according to the fiber strength equation $\sigma = F/A$ (F is the fracture load, A is the fiber cross-sectional area). Bearing this in mind, densification of fibers can not only reduce the spacing between individual CNT to increase the inter-tube interaction and the load transfer efficiency, but also effectively reduce the cross-sectional area of the derived CNTFs. Furthermore, a concomitant increase in orientation can be accomplished in part with a synergistic process of CNTFs densification and strengthening.

As per the existing literature, there is a limited number of studies addressing the process of densification to improve wet-spinning CNTFs. While those obtained from array spinning or direct dry spinning from FDCVD could be densified via applying exogenic force utilizing solvent-induced densification, mechanical compression, and multi-stage drafting. Thereby, we will give a concise description of these methods aiming to give hints on the densification enhancement of wet-spinning CNTFs.

Mechanical enhancement of CNTFs *via* the introduction of second mediator. As for wet-spinning fibers with highly aligned densely-packed microstructure, introducing an intermediate mediator to fill the voids inside the fibers is a more feasible approach. As illustrated in Figure S8a, a novel hybrid fiber with graphene oxide (GO) intercalating between CNTs was fabricated by Kim *et al.*[31] *via* simply blending different contents of GO with CNTs in CSA. Identified from small-angle X-ray scattering (SAXS) and wide-angle X-ray scattering (WAXS) analysis, the variation in microvoid structure were investigated. The voids exhibited a non-monotonous trend as the GO concentration increased. The voids length decreased first while increased dramatically above when GO ratio reach 20%. The specific density of fibers peaked with 10% GO decrease gradually in accordance with the change in voids volume fraction confirmed by X-ray microscopy (XRM). HR-TEM unveiled the paraphrased changes in microvoid structure *via* observing the fiber cross-sectional morphology

which that at low volume fraction. GO flake is monolayered and flexible enough to intercalate between CNTs while a multilayer GO featuring graphitic structure is separated from CNTs bundles with high bending stiffness. It could hinder the formation of an ideal structure in the equatorial direction in the CNTFs at a high-volume fraction. After incorporating of 10% GO, 53% increasing in tensile strength up to 6.05 $\pm$ 0.45 GPa and 42% increasing in modulus up to 422 $\pm$ 49 GPa were realized, respectively. This mechanical enhancement could be understood by virtue of equation (9) that Ω_2 augmented as the intra-fiber voids fulfilled with intercalated monolayer GO with generating additional bonding area. Coincidentally, CNTs/reduced GO (rGO) hybrid fibers with a high torsional strength with the ability to withstand a twist load were fabricated after meticulous modulation of spinning dope rheology, which showcases a lower porosity, closer interlayer distances and higher packing densities[32]. Excepting the low-dimension allotropes of CNTs with commensurate preeminent properties, graphene, conjugated polymers with rigid-rod morphology can also be incorporated into CNTFs to facilitate the load transfer between interfaces. CSA, the true thermodynamic solvent for CNTs which can also be protonated by various polymers containing aromatic hydrocarbons. They were employed to mitigate the incompatibility between CNTs and polyaromatic amide (PA) via forming hybridized lyotropic LC (Figure S8b)[33]. The seasoning of PA into CNTs dope propelled the fiber performance showcases chemical, mechanical and thermal robustness. Upon the addition of PA, the self-assembly process of CNTs during dispersion was regulated, and the local interfacial interaction forces induced the formation of LC domains as well as the orientation of CNTs. Hence, extensional flow can severely coerce tubes to be uniaxially oriented embodied in greater orientation factor by Raman spectroscopy and densely packed embodied in less porosity from 3.8 vol% for neat CNTFs to 0.4 vol% for hybrid PA/CNTFs (10/90) by XRM. Another hybrid CNTFs with modulated by PI exhibits high strength (4.8 $\pm$ 0.2 GPa), modulus (390 $\pm$ 48 GPa), and electrical conductivity (5.75 $\pm$ 0.84 MS m^{-1}) as a consequence of reduced voids volume and increased orientation factor[34].

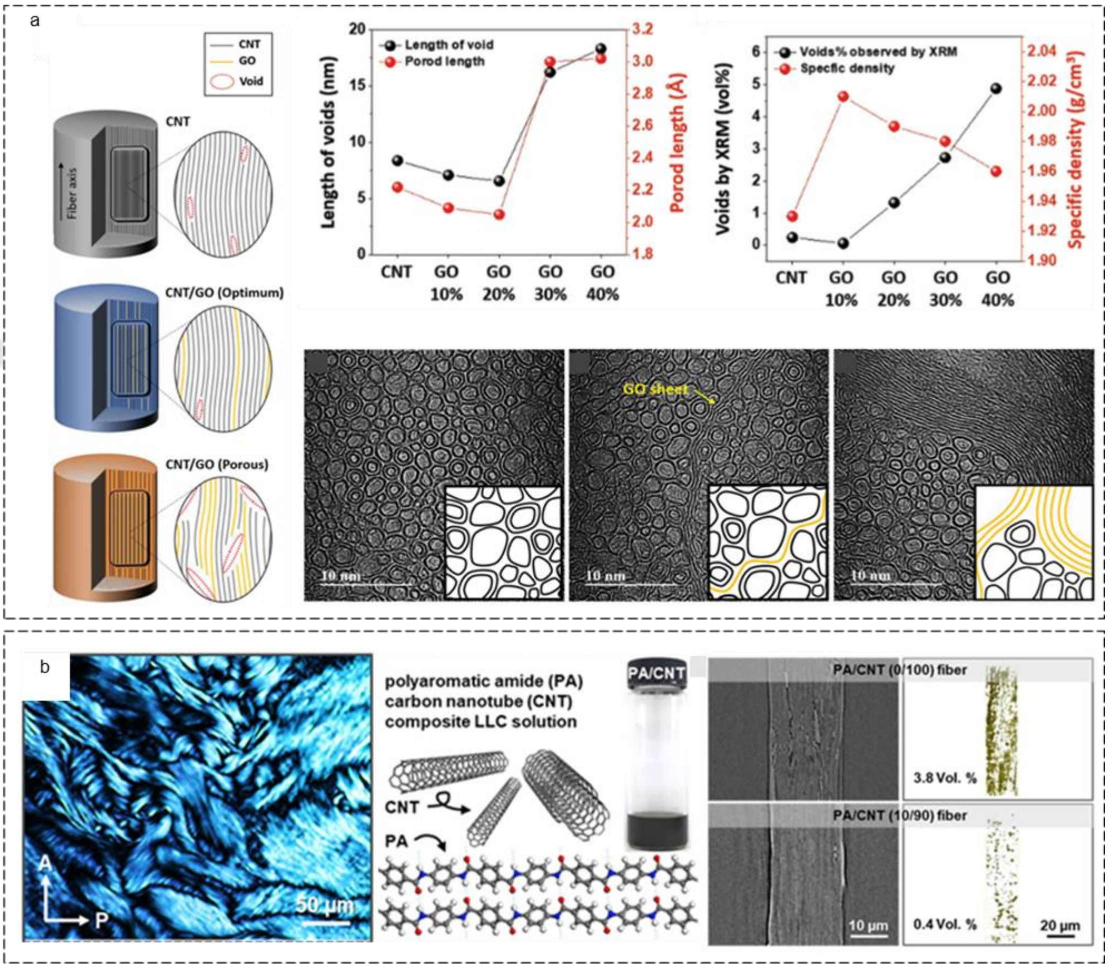

Figure S8. (a) Schematic illustrations of pristine CNTFs and CNTs/GO hybrid fibers and voids property variation after the addition of different concentrations of GO with corresponding HR-TEM images of cross sections of CNTFs. Adapted with permission from ref.[31]. Copyright 2022 John Wiley and Sons. (b) CNTs/PA fibers fabricated from hybridized lyotropic LC dopes with regulated phase behavior showing reduced voids volume. Adapted with permission from ref.[33]. Copyright 2022 Springer Nature.

Supplementary note 8. Strategies for enhancing electrical conductivity of CNTFs

Enhancing the electrical conductivity of CNTFs is divided into two aspects at this stage, one is from the perspective of wet-spinning process, which is mainly through the densification of CNTFs to promote the reduction of the inter-tube spacing of CNTs or the increase of the inter-tube pathway[35]. It could reduce the scattering of carriers and bring down the contact resistance. The highly oriented dense structure of CNTFs after various process adjustments in the strong acid system with p-type doping, could achieve a conductivity of 2.9-11.2 MS m^{-1}, with several orders of magnitude higher than that in other solvent systems[36-39]. In addition, as shown in Figure S9a and b, highly conductive CNTFs were prepared by introducing third-phase substances (polyaniline, graphene, *etc.*) to modulate the interfacial interactions between the nanotubes, enhancing the orientation of CNTFs, reducing the voids, and doping CNTs for fast electron transfer[40,41].

The other approach to increase the conductivity of CNTFs is to modulate the CNTs Fermi energy level to increase the density of hole or electron carriers by heteroatom doping. Behabtu *et al.*[4] developed iodine-doping approach to improve electrical conductivity, which was stabilized at 200 ℃ with a conductivity of 5 ±0.5 MS m^{-1}. Qiu *et al.*[42] immersed gold nanoparticles inside CNTs to achieve highly condensed electron densities near the Van Hove singularity, with inducing equivalent p-type doping. It was also found that surface-modified functional groups increased interfacial electron transport capability when CNTFs were acidified by immersion in HNO_3, with 95% electrical conductivity enhancement[43]. For the defective structure of CNTs, carbon atoms in the CNTs lattice are replaced by heterogeneous atoms for lattice doping, which introduces additional electrons or holes into the CNTs lattice, with modulating the carrier concentration. As shown in Figure S9c, Hwang *et al.*[36] used a combination of high-temperature thermal doping and plasma treatment to introduce boron and nitrogen atoms into the hexagonal carbon lattice of CNTs, thereby reducing

the jump or tunneling distance of electron transfer between CNTs. The derived CNTFs manifest a conductivity of 5896 S m^2 kg^{-1}.

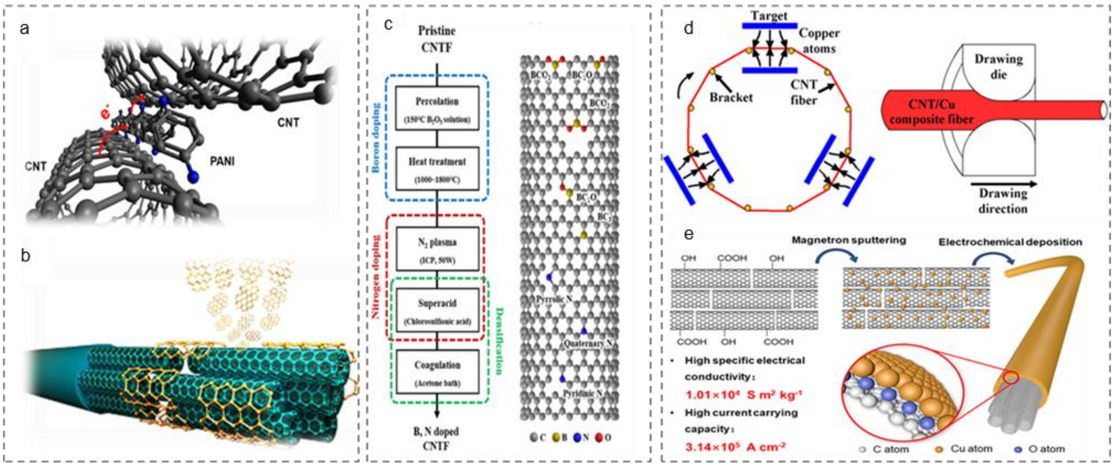

Figure S9. (a) The schematic illustration of electron tunneling or hopping into CNTs-PANI-CNTs pathway. Adapted with permission from ref.[41]. Copyright 2023 Elsevier. (b) Small, high-quality graphene can bridge adjacent CNTs, facilitating the formation of strong structures with rich conductive pathways. Adapted with permission from ref.[40]. Copyright 2023 American Chemical Society. (c) Schematic representations of boron, nitrogen doping and densification of CNTFs and bonding configurations of introduced boron and nitrogen on the sidewall of the CNTs. Adapted with permission from ref.[36]. Copyright 2021 Elsevier. (d) Schematic of physical vapor deposition of copper on the CNTFs, and drawing treatment processing for CNTs/Cu composite fiber. Adapted with permission from ref.[35]. Copyright 2017 Elsevier. (e) Schematic of coating a compact and uniform Cu shell on the surface of a wet-spinning CNTFs by a combination of magnetron sputtering and electrochemical deposition. Adapted with permission from ref.[44]. Copyright 2023 American Chemical Society.

CNTFs are homogeneously compounded with metals by electrodeposition, chemical deposition and magnetron sputtering to achieve the highly conductive fibers. For example, Tran *et al.*[45] first sputtered Au on the fiber surface *via* the electrodeposition method. After deposition of copper on the CNTFs, the CNTs/Au/Cu hybrid fiber displayed a tensile strength of 0.74 GPa and a conductivity up to 4.65×10^5 S cm^{-1}. Sue *et al*.[46] introduced sulfyl groups on the surface of the CNTFs by chemical deposition,

and chemically reduced them with copper on the surface to obtain strong interaction force. The CNT/Cu core-shell fibers exhibited an electrical conductivity of 3.6×10^7 S m^{-1}, tensile strength of 1 GPa and capacitance of 1.04×10^5 A cm^{-2} were obtained. Han *et al.* adopted a magnetron sputtering method to develop metal/CNT hybrid fiber, which similar to the physical vapor phase precipitation (PVD) method. As shown in Figure S9d, metal particles were deposited on the surface of CNTFs with the effect of electric field force[35]. Subsequently, in 2019, Wang *et al.*[39] combined with the vacuum thermal diffusion process to spread copper and aluminum metal atoms into the interior of the CNTs, with enhancing the friction between CNTs by ‘pinning and bridging’. The tensile strength and the modulus of the resultant hybrid CNTFs are 6.6 GPa and 500 GPa, respectively, while maintaining a high electrical conductivity of 1.8×10^7 S m^{-1}. However, Liu *et al.*[38] plated dense and uniform Cu shells on the surface of wet-spinning CNTFs by magnetron sputtering, with the assistance of electrochemical deposition to make the oxygen-containing functional groups on the surface to strengthen the interact with the sputtered Cu atoms (Figure S9e). The as-prepared hybrid CNTFs manifests an ultra-high specific electrical conductivity of $1.01 \pm 0.04 \times 10^4$ S m^2 kg^{-1}, which is even 56% higher than that of copper. The current carrying capacity of the thus-fabricated CNTFs reaches 3.14×10^5 A cm^{-2}, which is three times of that commercial copper wires.

Supplementary note 9. Strategies for enhancing thermal conductivity of CNTFs

Improving the density and orientation of CNTFs were achieved through the wet-spinning process, which reduced inter-tube spacing and enhanced thermal conduction at the tube interfaces. Ku *et al.*[28] controlled the wet-spinning process to achieve an increase in density and alignment, thereby maximizing the performance of the fibers. The fibers fabricated were highly oriented, which demonstrated a thermal conductivity of 398 $\pm$ 27 W m^{-1} K^{-1}. In addition, Ku *et al.*[47] also assembled highly oriented SWCNTs and MWCNTs bundles into CNTFs, which achieved a high packing density of 1.92 g cm^{-3}. Afterwards, they delved into the impact of heat treatment temperature on the internal crystalline structure and thermal conductivity of the derived CNTFs. Compared with CNTFs comprised of SWCNTs, there is a coupling effect of stacking between single-walled and multi-walled tubes that can form a tightly interconnected network of graphite domains. The highly ordered arrangement and high longitudinal crystallinity reduce phonon scattering, thus enhancing the thermal conductivity. Subsequently, the team introduced the secondary medium polyimide (PI) to fill the voids to enhance the packing density of CNTFs. After carbonization and graphitization at the optimal ratio of PI (30%) to CNTs, they achieved a thermal conductivity of 496 $\pm$ 38 W m^{-1} K^{-1}[34].

Qiu *et al.*[43] realized densification by using local electrostatic cohesion and the local coulomb electrostatic cohesion between CNTs bundles caused by surface dipole moments after densification treatment with EG and HNO_3. Thermal conductivity increased from 21.2 to 30.5 and 37.2 W m^{-1} K^{-1}, respectively. The thermal conductivity was increased from 30.5 to 50 W m^{-1} K^{-1} by introducing gold nanoparticles to induce the coupling effect of low-frequency phonon vibrations between CNTs[42]. Attempts were also made to introduce halogenated iodine molecules to form $(I_3)^-$ and $(I_5)^-$ polyiodide chains as additional heat transfer channels, which induced low-frequency phonons from the interfacial carbon atoms and the thermal conductivity of the fibers from 30.5 to 47.3 W m^{-1} K^{-1} (about 55%)[48]. Through high temperature thermal

reduction and ultra-high temperature graphitization, oxygen-containing functional groups can be further removed[49-51]. While structural parameters such as crystallinity, crystal zone size and orientation of CNTFs can be improved, multistage structural defects within the fibers can be reduced to obtain CNTFs with high thermal conductivity[34,47].

Table S1. Methodologies for the characterization and the performance evaluation.

Aggregation state	Property	Method
CNTs	Aspect ratio	AFM, TEM, Shear an extensional viscosity
	Defect	Raman spectroscopy, TEM
	Impurity	TGA, EDS, SEM
	Functionalization	XPS, FTIR, Raman
CNTs dispersion	Dispersion state	Shear viscosity, POM, SANS, SAXS, cryo-EM
CNTFs	Mechanics	Universal testing machine
	Electrical conductivity	Kelvin Four-terminal sensing
	Thermal conductivity	The 3ω method, Steady-state dc thermal bridge method, T-type method

References:

1. Jiang X, Qu S, Shao Z *et al.* Effect of dispersion time on the microstructural and mechanical properties of carbon nanotube solutions and their spun fibers. *Compos Commun* 2021; **27**: 100872.
2. Haggenmueller R, Rahatekar SS, Fagan JA *et al.* Comparison of the quality of aqueous dispersions of single wall carbon nanotubes using surfactants and biomolecules. *Langmuir* 2008; **24**: 5070-5078.
3. Wu X, Mukai K, Asaka K *et al*. Effect of surfactants and dispersion methods on properties of single-walled carbon nanotube fibers formed by wet-spinning. *Appl Phys Express* 2017; **10**: 055101.
4. Behabtu N, Young CC, Tsentalovich DE *et al.* Strong, light, multifunctional fibers of carbon nanotubes with ultrahigh conductivity. *Science* 2013; **339**: 182-186.
5. Maillaud L, Headrick RJ, Jamali V *et al.* Highly concentrated aqueous dispersions of carbon nanotubes for flexible and conductive fibers. *Ind Eng Chem Res* 2018; **57**: 3554-3560.
6. Bazbouz MB, Aziz A, Copic D *et al.* Fabrication of high specific electrical conductivity and high ampacity carbon nanotube/copper composite wires. *Adv Electron Mater* 2021; **7**: 2001213.
7. Wang P, Kim M, Peng Z *et al.* Superacid-surfactant exchange: enabling nondestructive dispersion of full-length carbon nanotubes in water. *ACS Nano* 2017; **11**: 9231-9238.
8. Zhang S, Koziol KKK, Kinloch IA *et al.* Macroscopic fibers of well-aligned carbon nanotubes by wet spinning. *Small* 2008; **4**: 1217-1222.
9. Lan M, Jia X, Tian R *et al.* Highly redispersible CNT dough for better processiblity. *J Mater Sci Technol* 2023; **152**: 65-74.
10. Razal JM, Gilmore KJ and Wallace GG. Carbon nanotube biofiber formation in a polymer-free coagulation bath. *Adv Funct Mater* 2008; **18**: 61-66.
11. Barisci JN, Tahhan M, Wallace GG *et al.* Properties of carbon nanotube fibers spun from DNA-stabilized dispersions. *Adv Funct Mater* 2004; **14**: 133-138.

12. Moulton SE, Maugey M, Poulin P *et al.* Liquid crystal behavior of single-walled carbon nanotubes dispersed in biological hyaluronic acid solutions. *J Am Chem Soc* 2007; **129**: 9452-9457.

13. Horn DW, Ao G, Maugey M *et al.* Dispersion state and fiber toughness: antibacterial lysozyme-single walled carbon nanotubes. *Adv Funct Mater* 2013; **23**: 6082-6090.

14. Nyankima AG, Horn DW and Davis VA. Free-standing films from aqueous dispersions of lysozyme, single-walled carbon nanotubes, and polyvinyl alcohol. *ACS Macro Lett* 2013; **3**: 77-79.

15. Ramesh S, Ericson LM, Davis VA *et al.* Dissolution of pristine single walled carbon nanotubes in superacids by direct protonation. *J Phys Chem B* 2004; **108**: 8794-8798.

16. Chen J, Hamon MA, Hu H *et al.* Solution properties of single-walled carbon nanotubes. *Science* 1998; **282**: 95-98.

17. Minami N, Kazaoui S, Jacquemin R *et al.* Optical properties of semiconducting and metallic single wall carbon nanotubes: effects of doping and high pressure. *Synthetic metals* 2001; **116**: 405-409.

18. Zhou W, Fischer JE, Heiney PA *et al.* Single-walled carbon nanotubes in superacid: X-ray and calorimetric evidence for partly ordered H_2SO_4. *Phys Rev B* 2005; **72**: 045440.

19. Khokhlov AR. Theories based on the Onsager approach. *Liquid crystallinity in polymers*, 1991; 97-129.

20. Davis VA, Ericson LM, Parra-Vasquez ANG *et al.* Phase behavior and rheology of SWNTs in superacids. *Macromolecules* 2004; **37**: 154-160.

21. Kirkwood JG and Auer PL. The visco-elastic properties of solutions of rod-like macromolecules. *J Chem Phys* 1951; **19**: 281-283.

22. Rai PK, Pinnick RA, Parra-Vasquez ANG *et al.* Isotropic-nematic phase transition of single-walled carbon nanotubes in strong acids. *J Am Chem Soc* 2006; **128**: 591-595.

23. Flory PJ. Phase equilibria in solutions of rod-like particles. *Proc Math Phys Eng*

Sci 1956; **234**: 73-89.

24. Jamali V, Mirri F, Biggers EG *et al.* Enhanced ordering in length-polydisperse carbon nanotube solutions at high concentrations as revealed by small angle X-ray scattering. *Soft Matter* 2021; **17**: 5122-5130.
25. Yilmaz L and McHugh AJ. Analysis of nonsolvent–solvent–polymer phase diagrams and their relevance to membrane formation modeling. *J Appl Polym Sci* 1986; **31**: 997-1018.
26. Paul DR. Diffusion during the coagulation step of wet-spinning. *J Appl Polym Sci* 1968; **12**: 383-402.
27. Bucossi AR, Cress CD, Schauerman CM *et al.* Enhanced electrical conductivity in extruded single-wall carbon nanotube wires from modified coagulation parameters and mechanical processing. *ACS Appl Mater Interfaces* 2015; **7**: 27299-27305.
28. Kim SG, Choi GM, Jeong HD *et al.* Hierarchical structure control in solution spinning for strong and multifunctional carbon nanotube fibers. *Carbon* 2022; **196**: 59-69.
29. Jeong HD, Kim SG, Choi GM *et al.* Theoretical and experimental investigation of the wet-spinning process for mechanically strong carbon nanotube fibers. *Chem Eng J* 2021; **412**: 128650.
30. Fan Z, Cao M, Yang WB *et al.* The evolution of microstructure and thermal conductivity of mesophase pitch-based carbon fibers with heat treatment temperature. *New Carbon Mater* 2019; **34**: 38-43.
31. Kim SG, Heo SJ, Kim JG *et al.* Ultrastrong hybrid fibers with tunable macromolecular interfaces of graphene oxide and carbon nanotube for multifunctional applications. *Adv Sci* 2022; **9**: 2203008.
32. Eom W, Lee E, Lee SH *et al.* Carbon nanotube-reduced graphene oxide fiber with high torsional strength from rheological hierarchy control. *Nat Commun* 2021; **12**: 396.
33. Ryu K-H, Kim J-G, Lee D *et al.* Boost up the mechanical and electrical property of CNT fibers by governing lyotropic liquid crystalline mesophases with aramid

polymers for robust lightweight wiring applications. *Adv Fiber Mater* 2022; **5**: 514-526.

34. Kim SG, Heo SJ, Kim S *et al.* Ultrahigh strength and modulus of polyimide-carbon nanotube based carbon and graphitic fibers with superior electrical and thermal conductivities for advanced composite applications. *Compos B: Eng* 2022; **247**: 110342.
35. Han B, Guo E, Xue X *et al.* Fabrication and densification of high performance carbon nanotube/copper composite fibers. *Carbon* 2017; **123**: 593-604.
36. Hong S, Nam J, Park S *et al.* Carbon nanotube fibers with high specific electrical conductivity: Synergistic effect of heteroatom doping and densification. *Carbon* 2021; **184**: 207-213.
37. Sun H, Zhang Y, Zhang J *et al.* Energy harvesting and storage in 1D devices. *Nat Rev Mater* 2017; **2**: 1-12.
38. Jiao X, Shi C, Zhao Y *et al.* Efficient fabrication of high-quality single-walled carbon nanotubes and their macroscopic conductive fibers. *ACS Nano* 2022; **16**: 20263-20271.
39. Wang GJ, Cai YP, Ma YJ *et al.* Ultrastrong and stiff carbon nanotube/aluminum–copper nanocomposite via enhancing friction between carbon nanotubes. *Nano Lett* 2019; **19**: 6255-6262.
40. Li L, Sun T, Lu S *et al.* Graphene interlocking carbon nanotubes for high-strength and high-conductivity fibers. *ACS Appl Mater Interfaces* 2023; **15**: 5701-5708.
41. Lee D, Kim SG, Kim J *et al.* Highly conductive and mechanically strong metal-free carbon nanotube composite fibers with self-doped polyaniline. *Carbon* 2023; **213**: 118308.
42. Qiu L, Zou H, Wang X *et al.* Enhancing the interfacial interaction of carbon nanotubes fibers by Au nanoparticles with improved performance of the electrical and thermal conductivity. *Carbon* 2019; **141**: 497-505.
43. Qiu L, Wang X, Tang D *et al.* Functionalization and densification of inter-bundle interfaces for improvement in electrical and thermal transport of carbon nanotube fibers. *Carbon* 2016; **105**: 248-259.

44. Xu L, Jiao X, Shi C *et al.* Single-walled carbon nanotube/copper core-shell fibers with a high specific electrical conductivity. *ACS Nano* 2023; **17**: 9245-9254.
45. Tran TQ, Lee J K, Chinnappan A *et al.* Strong, lightweight, and highly conductive CNT/Au/Cu wires from sputtering and electroplating methods. *J Mater Sci Technol* 2020; **40**: 99-106.
46. Daneshvar F, Chen H, Zhang T *et al.* Fabrication of light-weight and highly conductive copper-carbon nanotube core-shell fibers through interface design. A*dv Mater Interfaces* 2020; **7**: 2000779.
47. Lee D, Kim SG, Hong S *et al*. Ultrahigh strength, modulus, and conductivity of graphitic fibers by macromolecular coalescence. *Sci Adv* 2022; **8**: eabn0939.
48. Qiu L, Zou H, Zhu N *et al.* Iodine nanoparticle-enhancing electrical and thermal transport for carbon nanotube fibers. *Appl Therm Eng* 2018; **141**: 913-920.
49. Li P, Liu Y, Shi S *et al*. Highly crystalline graphene fibers with superior strength and conductivities by plasticization spinning. *Adv Funct Mater* 2020; **30**: 2006584.
50. Ming X, Wei A, Liu Y *et al.* 2D-topology-seeded graphitization for highly thermally conductive carbon fibers. *Adv Mater* 2022; **34**: 2201867.
51. Li P, Wang Z, Qi Y *et al.* Bidirectionally promoting assembly order for ultrastiff and highly thermally conductive graphene fibres. *Nat Commun* 2024; **15**: 409.
